# Inferring regulatory element landscapes and transcription factor networks from cancer methylomes

**DOI:** 10.1186/s13059-015-0668-3

**Published:** 2015-05-21

**Authors:** Lijing Yao, Hui Shen, Peter W Laird, Peggy J Farnham, Benjamin P Berman

**Affiliations:** Norris Comprehensive Cancer Center, Keck School of Medicine, University of Southern California, 1450 Biggy Street, NRT 6503, Los Angeles, CA 90089-9601 USA; Center for Epigenetics, Van Andel Research Institute, Grand Rapids, MI 49503 USA; Bioinformatics and Computational Biology Research Center, Department of Biomedical Sciences, Cedars-Sinai Medical Center, AHSP Bldg., Suite A8111, Los Angeles, CA 90048 USA

## Abstract

Recent studies indicate that DNA methylation can be used to identify transcriptional enhancers, but no systematic approach has been developed for genome-wide identification and analysis of enhancers based on DNA methylation. We describe ELMER (Enhancer Linking by Methylation/Expression Relationships), an R-based tool that uses DNA methylation to identify enhancers and correlates enhancer state with expression of nearby genes to identify transcriptional targets. Transcription factor motif analysis of enhancers is coupled with expression analysis of transcription factors to infer upstream regulators. Using ELMER, we investigated more than 2,000 tumor samples from The Cancer Genome Atlas. We identified networks regulated by known cancer drivers such as GATA3 and FOXA1 (breast cancer), SOX17 and FOXA2 (endometrial cancer), and NFE2L2, SOX2, and TP63 (squamous cell lung cancer). We also identified novel networks with prognostic associations, including RUNX1 in kidney cancer. We propose ELMER as a powerful new paradigm for understanding the *cis*-regulatory interface between cancer-associated transcription factors and their functional target genes.

**Electronic supplementary material:**

The online version of this article (doi:10.1186/s13059-015-0668-3) contains supplementary material, which is available to authorized users.

## Background

ENCODE and other large-scale efforts have mapped transcription factor binding sites, histone modifications, and chromatin accessibility in a common set of cell lines [1, 2]. Integration of these genome-wide maps has led to the view that distinct epigenetic marks are not independent but rather that chromatin is organized into discrete functional states marked by particular combinations of individual features [3, 4]. Computational methods such as chromHMM [5] and Segway [6] have been developed to identify these states from individual histone and accessibility features, and the state most consistently linked to cellular identity is the ‘active enhancer’ state defined by the presence of histone H3 lysine 27 acetylation and low levels of the canonical promoter mark, H3 lysine 4 tri-methylation [5, 7, 8]. Active enhancers are enriched for sequences bound by cell-type specific transcription factors, reinforcing their preeminent role in encoding the cis-regulatory logic of the genome. Projects such as the NIH Roadmap [2, 9] and Blueprint [10] have also mapped histone modifications and chromatin accessibility in primary human tissues, identifying a large set of enhancers from many different cell types. Others have employed these datasets to identify large numbers of enhancer-promoter pairs in 12 human cell types [11, 12]. However, approaches such as ChIP-seq or DNAse hypersensitivity assays require careful tissue handling (to avoid protein degradation) and relatively large numbers of cells (10^6^ to 10^7^) and thus have not been applied to the identification of enhancers in primary tumor tissues.

Fortunately, enhancers can also be identified using patterns of 5-methylcytosine, an epigenetic mark that is maintained more stably than protein marks, and can be detected genome-wide in as few as 1,000 cells [13]. Historically, DNA methylation research has focused on gene promoter regions (reviewed in [14]). While early work suggested that DNA methylation could mark enhancer regions of interest [15], this was not widely appreciated until the first complete and unbiased study of DNA methylation in human cells revealed enhancer regions as being unmethylated in a cell-type specific manner [16]. A later study used the same whole-genome bisulfite sequencing (WGBS) approach to identify all genomic regions containing little or no methylation; these regions overwhelmingly corresponded to enhancers and other distal regulatory elements [17]. Cell-type specific demethylation of enhancers was confirmed by targeted bisulfite sequencing in the ENCODE project [1]. More recently, WGBS data from 30 diverse human cell types showed that enhancers had highly dynamic methylation patterns - roughly 30% of the most cell type-specific regions in the genome overlapped known enhancers (compared to 5% that overlapped gene promoters). The mechanism underlying these correlations is not well understood, but could involve de-methylation of DNA initiated by transcription factor binding ([17]; reviewed in [18]) and maintained by DNA methyltransferase protection by Histone H3 lysine 4 monomethyl groups [19].

In cancer tissues, recent studies have shown that cancer-specific enhancers and transcription factor binding sites can be identified from DNA methylation profiles. The first genome-scale analysis of transcription factor binding sites in cancer found that binding by transcription factors such as Sp1, NRF1, and YY1 could protect CpG island gene promoters from cancer-specific hypermethylation [20]. Our WGBS study of a human colon cancer identified all genomic regions that changed from a methylated state in the normal colon to an unmethylated state in the tumor; 90% of these regions overlapped known enhancers, and a highly disproportionate number contained binding sites for the AP-1 transcription factor [21]. A more recent study showed that DNA methylation changes at enhancer elements were significantly better than those at promoters for predicting gene expression changes of target genes in cancer [22]. WGBS was recently used to show that unmethylated regions were enriched for binding sites for subtype-specific transcription factors in pediatric medulloblastoma (LEF1 for the WNT subtype and GLI2 for the SHH subtype [23]).

Once an enhancer has been identified by DNA methylation, identification of the specific target gene or genes whose expression is modulated by that enhancer can be challenging because the target genes can be thousands to millions of base pairs away from the enhancer. A study using chromatin conformation sequencing (ChIA-PET) to study enhancer/promoter interactions found that the median distance between an enhancer and a promoter was approximately 50 kb, and that at least 40 % of enhancers skip one or more annotated genes to find their target promoter [24]. The ChIA-PET dataset was used in conjunction with DNA methylation and RNA-seq data from breast cancer cases in The Cancer Genome Atlas (TCGA) to identify enhancer/promoter pairs *in vivo* [25]. Other reports have also shown that methylation of distal regulatory sites is closely related to gene expression levels across the genome [26]. Here, we present a statistical framework for identification of cancer-specific enhancers and paired gene promoters, and use it to investigate approximately 3,000 cases from 11 tumors types in the TCGA ‘Pan Cancer’ analysis set [27]. Our R software package, ELMER, uses only methylation and expression data, and does not require any chromatin conformation or ChIP-seq data. Furthermore, by identifying transcription factor binding motifs present within enhancers, and incorporating expression patterns of upstream transcription factors, ELMER is able to infer transcription factor networks activated in specific cancer subtypes. This work suggests a general approach for identifying *in vivo* transcription factor networks and the associated regulatory control sequences altered in cancer.

## Results

### Identifying cancer-specific DNA methylation changes in distal enhancer regions for 10 cancer types

To identify cancer-specific changes in DNA methylation, we obtained 3,381 DNA methylation datasets for 11 types of primary tumors from the TCGA Pan Cancer analysis set [27]. The cancer types we included in our analyses were leukemia (LAML), lung adenocarcinoma (LUAD), lung squamous cell carcinoma (LUSC), kidney renal clear cell carcinoma (KIRC), bladder urothelial carcinoma (BLCA), uterine corpus endometrioid carcinoma (UCEC), glioblastoma (GBM), head and neck squamous cell carcinoma (HNSC), breast cancer (BRCA), colon adenocarcinoma (COAD), and rectal adenocarcinoma (READ). Based on previous TCGA studies [28], COAD and READ are very similar and are often combined for analyses. Therefore we combined these two cancer types (indicated herein as CRC), resulting in 10 different primary tumor types. The TCGA ID numbers for all samples can be found in Additional file 1.

**Fig. 1:**
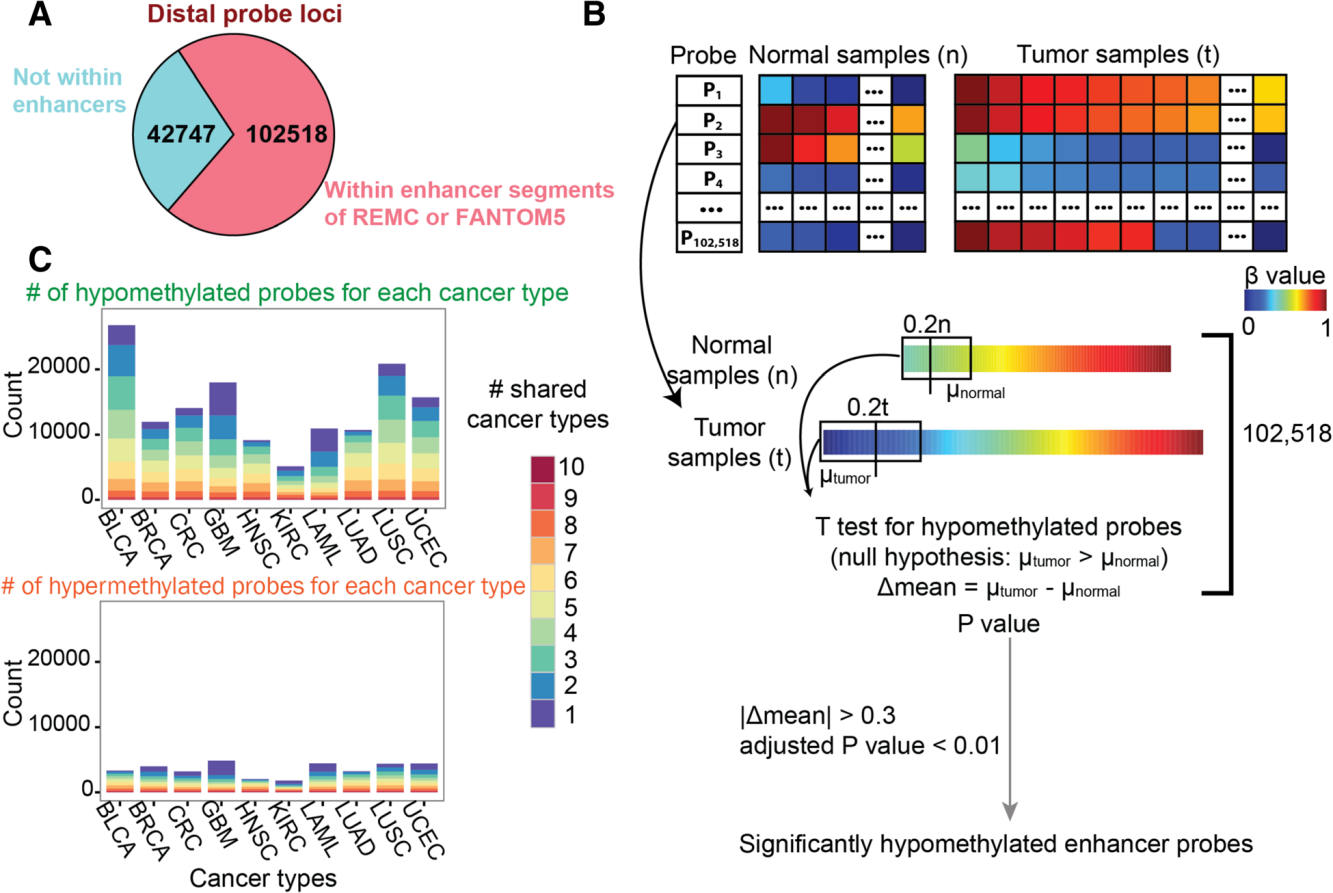
Identifying cancer-specific DNA methylation changes in distal enhancer regions. **a** Out of 145,265 distal probes on the HM450k platform, 102,518 were contained within our annotated enhancer regions (with approximately 1/8 of all distal enhancers being covered by at least one probe). **b** The statistical method used to identify probes hypomethylated (or hypermethylated) in cancer (see Methods for additional details). The heatmap in the top panel shows the DNA methylation level at each probe p_i_ for each sample from a particular cancer type (either an adjacent normal, or a tumor). Each cell is a methylation β value, reflecting the fraction of methylated DNA molecules at each CpG probe. The remainder of the panel illustrates our statistical test, which compares only the most extreme 20 % of normal samples to the most extreme 20 % of tumor samples, in order to identify probes hypomethylated in only a subset of tumors. (**c**) Shown is a histogram representing the number of cancer-specific hypomethylated (top graph) or hypermethylated (bottom graph) distal enhancer probes identified for each cancer type. The fraction of these probes shared by one or more other tumor types is indicated by the color bars (1 indicates that the probe is hypomethylated in only that tumor type, 2 indicates that it is hypomethylated in one other tumor type, and so on)

The DNA methylation datasets were produced using the Illumina Infinium HumanMethylation450 (HM450) BeadChip platform. The HM450 array allows the integration of more than 485,000 methylation sites at single-nucleotide resolution, covering 96 % of CpG islands and 99 % of RefSeq genes in the human genome. We used TCGA Level 3 data, which are normalized using platform-specific internal controls, and mask out probes for failure/SNP/repeats on the HumanMethylation450 array. Then, because we focused on distal enhancers, we selected only those probes that are greater than +/- 2 kb from a known TSS (defined using GENCODE v15 [29], resulting in a set of 145,265 distal probes. We next wanted to limit the number of candidate probes tested, so we filtered based on two large enhancer databases. While these databases do not include a large number of primary tumors, they do include cancer cell lines and a large number of cell types. The largest enhancer set came from a combination of enhancers from the Roadmap Epigenomics Mapping Consortium (REMC) and the Encyclopedia of DNA Elements (ENCODE) Project, in which enhancers were identified using ChromHMM [30] for 98 tissues or cell lines [2, 9, 31]. We used the union of genomic elements labeled as EnhG1, EnhG2, EnhA1, or EnhA2 (representing intergenic and intragenic active enhancers) in any of the 98 cell types, resulting in a total of 389,967 non-overlapping enhancer regions. A total of 101,918 distal probes from the HM450 array overlapped with these enhancer regions. We also downloaded from FANTOM5 enhancers having associated eRNAs for 400 distinct cell types [32]. The set of FANTOM5 enhancers (43,011) was much smaller than the set of REMC/ENCODE enhancers and only added an additional 600 probes, resulting in a total of 102,518 distal probe regions that overlapped with a previously identified enhancer region (Fig. 1a). This set of 102,518 distal enhancer probes (Additional file 2) included at least one CpG for 15 % of all enhancers in our annotation set, suggesting that the HM450k array can be used to sample a meaningful subset of enhancers genome-wide. It also included the majority (70 %) of all 145,265 distal probes on the array, so we believe that the analysis described below covers the vast majority of identifiable enhancers based on the HM450k array design. The ELMER R package also allows a complete search of all distal probes on the array, without filtering out the 30 % not associated with any known enhancer.

To identify enhancers that displayed cancer-specific changes in DNA methylation, we applied a *t*-test to identify enhancer probes that were significantly hypermethylated or hypomethylated within tumor samples of each cancer type, relative to TCGA adjacent normal samples from the same tissue (Fig. 1b; see Methods for details); a list of the identified hypermethylated or hypomethylated enhancer probes for each tumor type can be found in Additional file 3. We identified many more hypomethylated enhancer probes than hypermethylated probes for each of the 10 cancer types (Fig. 1c). Interestingly, most of the probes showing DNA methylation changes were found to have similar changes in DNA methylation in more than one cancer type. However, some probes were uniquely hypermethylated or hypomethylated in only one of the 10 tumor types. We note that it is not possible for us to be certain that the adjacent tissues collected by TCGA correspond to the same cell type from which the cancer arose, and therefore some of these methylation changes may correspond to tissue-specific differences rather than changes arising in the cancer. However, these differentially methylated probes are only candidates, as the next steps of ELMER (described below) use differences across all normal and tumor tissues (of the same cancer type) to determine true regulatory interactions.

### Linking methylation-affected enhancers to gene expression

**Fig. 2:**
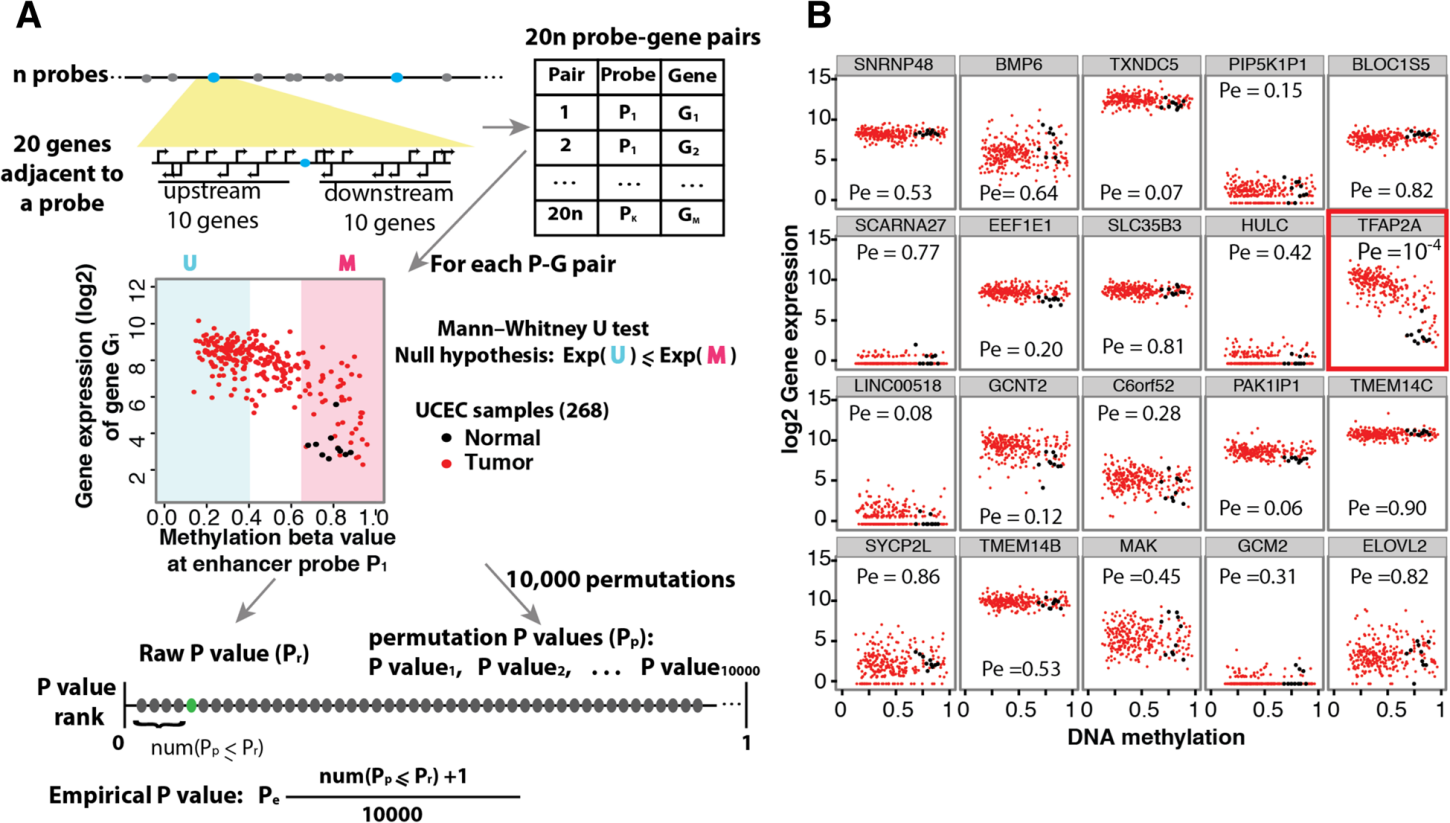
Linking differentially methylated probes to expression of nearby genes. **a** Shown is an illustration of the method used to associate each differentially methylated enhancer probe with one or more genes based on gene expression (see Methods section for additional details). For each of *n* probes identified as hypomethylated in a given cancer type (shown as blue circles), 10 genes upstream and 10 genes downstream were considered, yielding 20*n* statistical tests, one for each probe-gene pair. Each statistical test is performed across the complete set of normal and tumor samples *within* a particular cancer type. For instance, we show a scatterplot to illustrate such a test across the 258 endometrial (UCEC) tumor samples and 10 UCEC adjacent normals, showing the desired inverse correlation between methylation (x axis) and expression of the nearby gene (y axis). A Mann-Whitney *U* test was then performed, with the null hypothesis that the gene expression of group M samples is less or equal to that of group U samples. The U group consists of the 20 % least methylated samples for probe P_i_, and the M group consists of the top 20 % most methylated. The raw *P* value (*p*_*r*_) was compared to a permutation-based distribution of null *P* values, generated by performing 10,000 *U* tests between the actual gene G_j_ and DNA methylation a randomly selected distal non-enhancer probe. The empirical *p*_*e*_ value was calculated by the rank of *p*_*r*_ within the 10,000 trials. **b** Each scatter plot shows the methylation level of an example probe cg09606832 in all UCEC samples plotted against the expression of one of 20 adjacent genes. Only one gene, *TFAP2A*, shows a significant *p*_*e*_ indicating negative correlation, and is considered the linked gene

Although we identified approximately 100,000 enhancer probes that showed DNA methylation changes, it was not clear if all of these enhancers were actually involved in regulating gene expression. Previous studies have shown that only a portion of genomic regions classified as enhancers by chromatin marks or recruitment of histone acetyltransferases show activity in various assays [33, 34]. In addition, it is difficult to know which gene is regulated by each enhancer since enhancers can work from a distance, in either orientation, and do not necessarily regulate the closest gene. For example, in a ChIA-PET study using an antibody for RNA polymerase II, Li et al. [24] identified approximately 20,000 to 30,000 enhancer-promoter loops in MCF7 or K562 cells. Of these, more than 40 % of the enhancers skipped over the nearest gene to loop to a farther one. In order to identify target genes regulated by the distal regulatory elements, we analyzed expression data (RNA-seq) for 10 genes upstream and 10 genes downstream from each distal regulatory element; these 20 nearby genes constituted candidate gene targets. We preferred this method rather than those that evaluate all genes within a fixed-length genomic window, because the statistical power is controlled for the large degree in variation in gene density across the genome. Because not all TCGA samples had matched gene expression datasets, we selected the 2,841 TCGA samples that had matched gene expression (RNA-seq) and HM450k DNA methylation data (in Additional file 1). Although we realize that this method cannot identify target genes that are farther than ten genes away or on different chromosomes, we anticipated that many of the enhancers would regulate a gene within this distance [5]. Genes that are positively regulated by the enhancers should show a negative correlation between the DNA methylation level of the probe and expression of a putative target gene. We identified statistically significant CpG probe-gene pairs by comparing expression of the candidate gene in the upper vs. the lower quintile of samples, as measured by enhancer probe methylation. For this and all other downstream analyses, we included both normal and tumor samples, and only included samples within an individual cancer type (for example, UCEC), to avoid effects of tissue-specific differences and potential batch effects. We did not explicitly require expression changes between normal and tumor samples, because the number of normal samples with expression data were often quite limited. However, most genes identified did in fact show expression changes in the expected direction (downregulated for hypermethylated enhancers, and upregulated for hypomethylated enhancers; see the ‘tumor vs. normal expression’ worksheet in Additional file 4). To compare methylation quintiles vs. expression, we used a non-parametric *U* test, calculating an empirical *P* value using randomly assigned permutations of the methylation probe tested, and kept all pairs with an empirical *P* value <0.001 (Fig. 2a; see Methods for details). An example of one probe and its relationship to the expression of the 20 nearby genes in UCEC is shown in Fig. 2b. In this case, the probe showed an inverse correlation of methylation with expression of *TFAP2A*, which was the nearest gene upstream of the probe (approximately 7 kb away). A list of all putative enhancer-gene interactions can be found in Additional file 4.

**Fig. 3:**
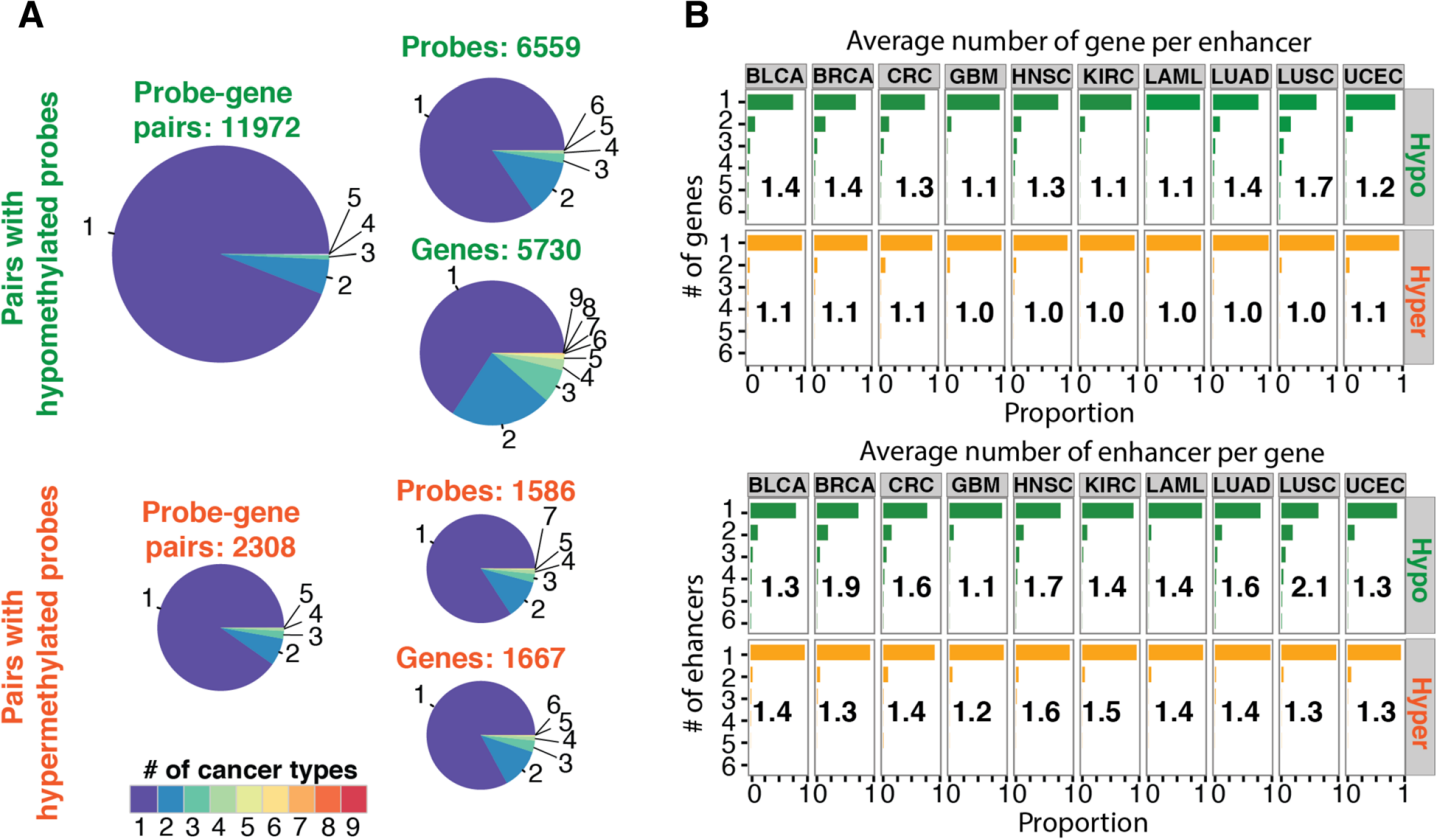
Comparison of probe-gene pairs between the different cancer types. **a** For the hypomethylated (top) and hypermethylated (bottom) probe-gene pairs, shown are pie charts that indicate the percentage of probe-gene pairs, probes, and genes that are present in one (purple) or shared by more than one of the 10 cancer types. **b** Using all probe-gene pairs, the distribution of the number of genes per enhancer (top) and the number of enhancers per gene (bottom) is shown for each individual cancer type. The mean of each is shown as a number within the bar plot

Using this method, we identified a total of 11,972 hypomethylated probe-gene pairs and 2,308 hypermethylated probe-gene pairs in the set of 10 tumor types (Fig. 3a), with the number of hypomethylated probe-gene pairs ranging from 499 to 3,847 in different tumor types, and the number of hypermethylated probe-gene pairs ranging from 119 to 464 (see Additional file 5 for a breakdown by type). Analysis of the probe-gene pairs revealed that most of the identified pairs were only found in one cancer type, suggesting that each enhancer regulates a specific gene in a tumor type-specific manner (Fig. 3a). Because some enhancers contained two or more probe features, we clustered probes that were within 500 bp of each other into 6,068 hypomethylated and 1,288 hypermethylated enhancer regions. Each enhancer was associated with an average of 1.0 to 1.7 genes, depending on tumor type, and each gene was associated with an average of 1.2 to 2.1 enhancers (Fig. 3b). Our work is consistent with previous studies indicating that distal elements commonly loop to or are associated with expression from 1 to 3 promoters [35]. Although the enhancer-gene pairs that we identified were highly specific for a certain tumor type, we found that approximately 34 % of the genes identified as regulated by a hypomethylated probe and approximately 17 % of the genes identified as regulated by a hypermethylated probe were targets in more than one tumor type (Fig. 3a), suggesting that a gene could utilize different enhancers in different tumor types for cancer-specific regulation.

**Fig. 4:**
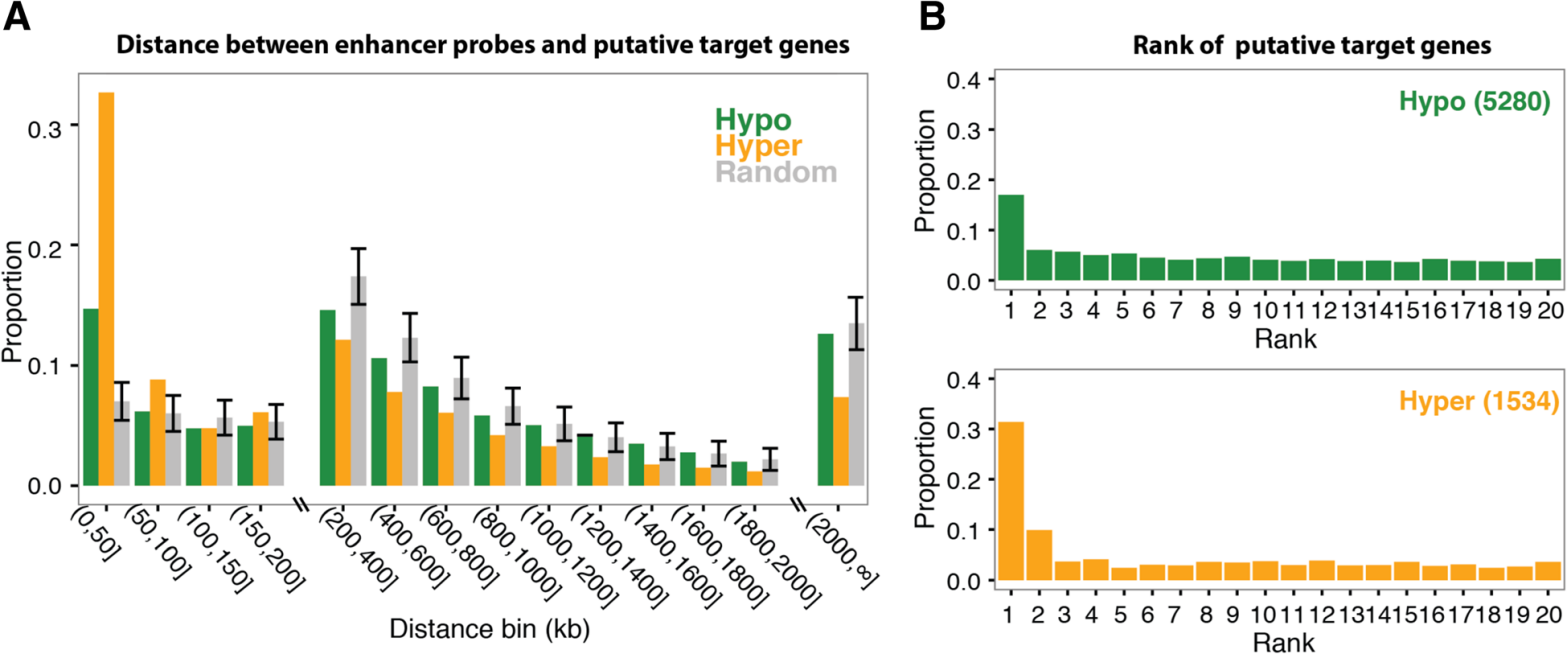
Physical characteristics of the probe-gene pairs. **a** A histogram of probe-gene distances for all pairs with a hypomethylated (green) or hypermethylated (yellow) probe. Shown is the distribution of the distance between linked distal enhancer probes and genes. The X-axis shows distances in bins of 50 kb or 200 kb. The Y-axis shows the proportion of all probe-gene pairs in the category (hyper- or hypomethylated) that fall into each range. These were compared to randomized datasets (gray bars), which were generated by randomly selecting 1,000 probes from the full set of 145,265 distal probes, and randomly pairing each with one of its 20 adjacent genes. We generated 1,000 such datasets to generate 95 % confidence intervals for each bin (+/-1.96* SD). **b** For each probe in a probe-gene pair, the 20 adjacent genes were ranked by distance, and shown is the proportion of all probes linked to genes of a given rank. For this analysis, probes linked to more than one gene and multiple probes linked to the same gene, were omitted

To further investigate the relationships between putative enhancers and linked target genes, we determined the frequency with which the probe-gene pairs we identified were separated by specific distances using window sizes of 50 or 200 kb (Fig. 4a). We found that both hypomethylated and hypermethylated probe-gene pairs were more frequent than random in the first 50 kb window, with hypermethylated pairs more dramatically so. A previous study using HiC to identify promoter-enhancer loops found that approximately 25 % of enhancer-promoter pairs were within a 50 kb range and approximately 75 % spanned 100 kb or larger genomic distance, with a median distance of 124 kb [36], whereas a recent study using *in situ* HiC identified contact domains ranging in size from 40 kb to 3 Mb, with a median size of 185 kb [37]. We then selected the set of probe-gene pairs where a single enhancer was only linked to a single gene (the great majority), and determined how often the linked gene corresponded to the nearest TSS. In previous studies, enhancers have been shown to loop to the nearest promoter only 27 % to 40 % of the time, skipping over the nearest TSS to loop to promoters farther away [24, 35]. We found that only approximately 15 % to 30 % of the time did the correlated gene correspond to the nearest TSS, with the percentage being higher for hypermethylated probe-gene pairs than for hypomethylated probe-gene pairs (Fig. 4b). This was significantly higher than the frequency of an enhancer being linked any other farther away gene (4 % to 8 %); because there was no selection for our statistical test to link to the nearest gene, the disproportional number of first-gene linkages gave us confidence that many or most of our linkages were true cis-regulatory links, including those that linked to more distant genes. If the linked gene did not correspond to the nearest TSS, there was very little preference to link to a nearby gene; the one exception was that hypermethylated enhancers were more likely to link to either the closest or second closest gene. This analysis is shown individually for each of the 10 tumor types in Additional file 6.

**Fig. 5:**
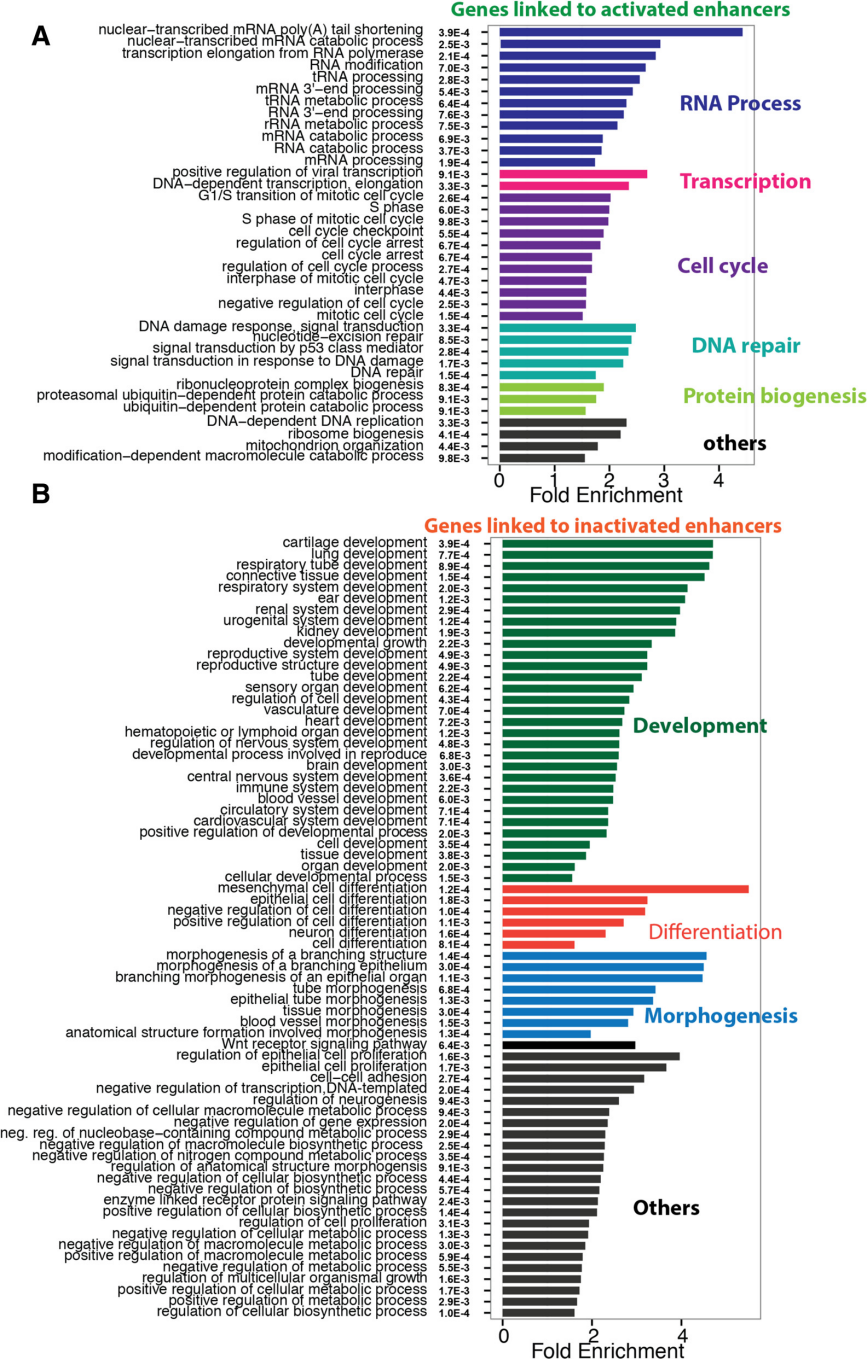
Gene Ontology (GO) enrichment analysis for genes identified in more than one cancer type. All genes identified in more than one cancer type by probe-gene pairs were analyzed for enrichment in particular GO categories, using the TopGO program. Activated genes (associated with hypomethylated enhancer probes) are shown in (**a**) and inactivated genes (associated with hypermethylated enhancer probes) are shown in (**b**). All GO categories with an adjusted enrichment *P* value of less than 0.01 (indicated next to the category name) and fold change more than 1.5 are included in the figure, and categories within the same biological process (color) are ordered by enrichment fold change (shown on the x axis). The adjusted enrichment *P* values are labeled in white in the graph

As indicated above, many of the genes that we identified as linked to enhancers with cancer-associated DNA methylation differences were actually identified in more than one cancer type, suggesting that they may have some common function in tumor initiation or progression. We selected all genes linked to an enhancer probe in more than one cancer type and performed a Gene Ontology enrichment analysis (Fig. 5). The 1,959 genes linked to hypomethylated (activated) enhancer probes correspond to genes upregulated in cancer, and the 284 genes linked to hypermethylated (inactivated) probes correspond to genes downregulated in cancer. Interestingly, we found that genes linked to hypermethylated (inactivated) enhancers were genes involved in development and differentiation. In contrast, genes linked to hypomethylated (activated) enhancers were classified as involved in the cell cycle and other cellular processes. Accordingly, we have identified known tumor suppressors (for example, *TSG1*, *RBM6*, *SPRY2*, *CDKN1A*, and *UBE4B*) in the set of genes potentially regulated by the hypermethylated enhancers and known oncogenes and cancer-associated genes (for example, *MYC*, *TERT*, *ERBB3*, *ERBB4*, *FGFR3*, *VEGFA*, *CDK7*, and *CCND1*) in the set of genes potentially regulated by the hypomethylated enhancers.

### Identification of regulatory TFs in each cancer type

**Fig. 6:**
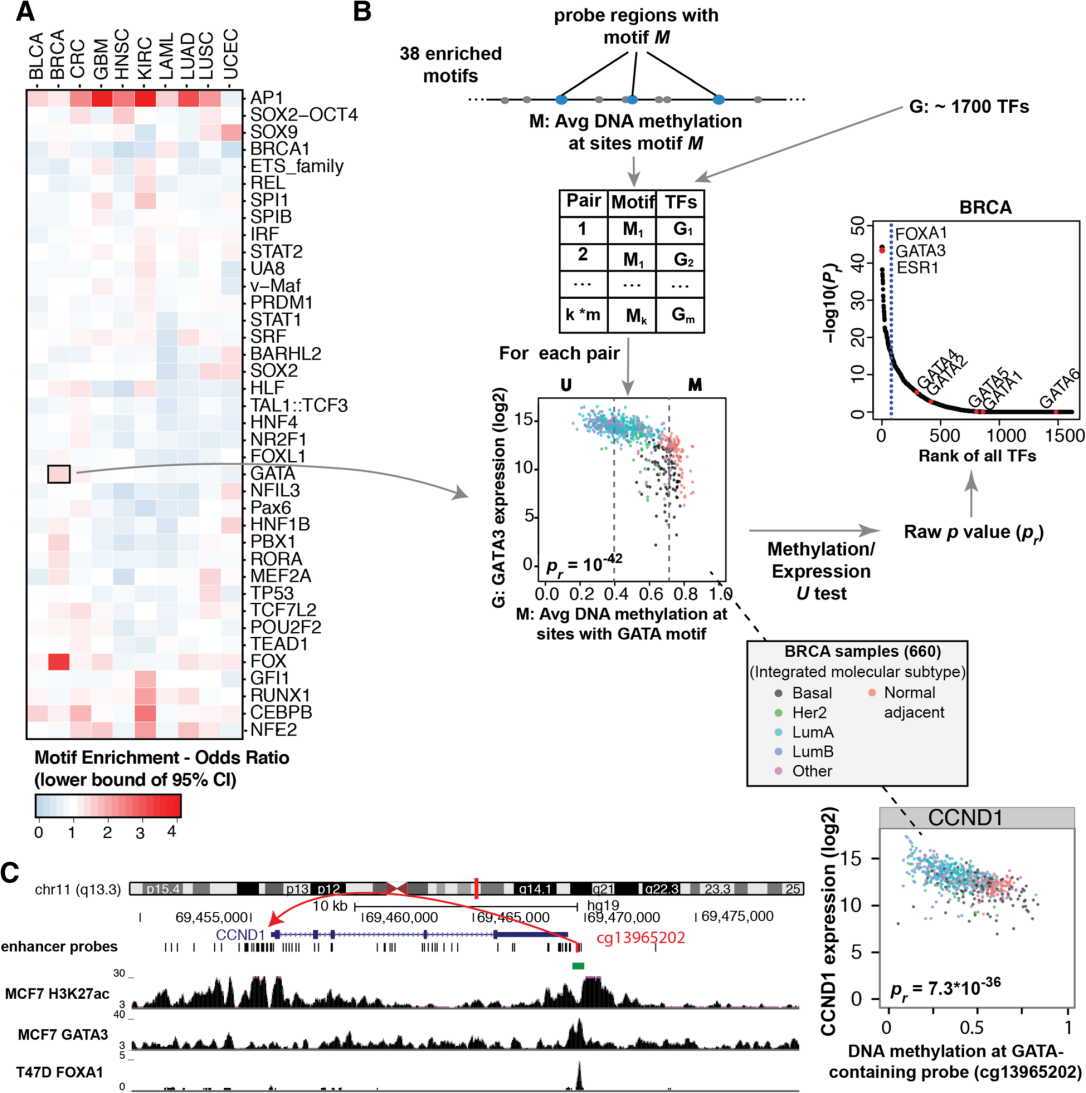
Identification of enhancer sets predicted to be co-regulated by the same transcription factor. **a** For 38 motifs enriched within hypomethylated probe-gene pairs in one or more cancer types, we calculated the 95 % confidence interval (CI) for the motif enrichment odds ratio; the lower bound of the 95 % CI is shown for each cancer type in the heatmap. **b** An illustration of the method for linking sets of enhancers with the same motif to an upstream TF regulator (see Methods for additional details). For each of the 38 (*m*) enriched motifs identified in panel (**a**), the average DNA methylation at all distal enhancer probes having that motif (in a specific tumor type), was compared to the expression levels of each of 1,777 (*k*) human TFs (Additional file 17). One such pair is shown as a scatter plot of all breast cancer (BRCA) tumor and adjacent normal samples, for the GATA motif and the GATA3 TF. BRCA samples (660) are color coded by integrated molecular subtypes defined by the TCGA Pan Cancer project, and extremes are selected as the 20% of samples with the lowest methylation (U) and the 20% with the highest methylation (M). A Mann-Whitney *U* test was performed to obtain the raw *P* value (*p*_*r*_). All 1,777 TFs were then ranked by *p*_*r*_ (plot at upper right), and the top 5% of the ranked TFs (dashed blue line) were considered to be significantly associated. The top three ranked TFs, along with each member of the specific DNA-binding family (in this case, GATAs) are labeled. Additional file 10 contains ranked TF plots for all motifs and all cancer types. **c** One of the 230 hypomethylated probe-gene pairs in BRCA containing a GATA motif corresponds to a downstream enhancer of the CCND1 gene. ENCODE ChIP-seq data in the Luminal-subtype MCF7 cell line verify that this enhancer is bound by the ELMER-predicted GATA3 TF

Changes in methylation status of an enhancer region can be due to gain (for hypomethyated enhancers) or loss (for hypermethylated enhancers) of site-specific transcription factors. To obtain insight into which site-specific TFs may be involved in setting the tumor-specific DNA methylation patterns, we examined the correspondence between cancer-specific hypermethylated or hypomethylated probes and known regulatory factor recognition sequence motifs. We used a combined set of motifs present in the JASPAR-Core [38] and FactorBook [39] datasets. We selected the enhancer probes that were identified in probe-gene pairs (using a cutoff of 0.001), then used the +/-100 bp sequence around each probe to search for instances of the 145 transcription factor motifs. We calculated the frequency of each motif within the hypomethylated (or hypermethylated) probe set for a given cancer vs. the frequency of the motif within the entire enhancer probe set. An odds ratio (OR) was calculated from these two frequencies, and only those motifs with an OR greater than 1.1 (at a confidence interval of 95 %) were selected as enriched within the given cancer type (motifs with less than 10 instances within the given probe set were excluded). All enriched motifs are listed in Additional file 7. For hypermethylated loci, we found that many of the identified motifs (such as E2F, EGR1, NRF1, Sp1) were associated with promoter regions (Additional file 8), suggesting that many of the hypermethylated loci may actually correspond to previously uncharacterized promoter regions. This likely accounts for the relatively high percentage of hypermethylated probe-pairs that showed linkage to the nearest annotated gene (Fig. 4b), which could reflect RNA-seq tags from the unannotated transcript isoform. Because many of the hypermethylated cases might not represent true distal enhancers, and because some may in fact be the result of cancer-related CpG Island promoter hypermethylation [14], we focused the remaining analyses on the 38 motifs found to be enriched within hypomethylated loci (Fig. 6a). Some of these motifs were common to various different cancers, such as AP1, which was enriched within nine of the 10 cancer types. Many motifs were more enriched in two or more specific tumor types, while others were limited to a single type, such as of GATA in BRCA, TP53/TP63 in LUSC, and HNF1A/B in UCEC.

Different members of a TF family have very similar DNA binding domains that can bind very similar or identical motifs. For example, we have previously shown that GATA1 and GATA2 bind to the same regulatory regions [40] and that members of the E2F family can bind to the same promoters [41]. Thus, identification of a motif does not uniquely identify the TF that binds *in vivo* to a region containing that motif. However, there is evidence to support the hypothesis that expression levels of a particular TF can correlate with levels of demethylation and subsequent gene expression [18, 42, 43]. To discover which members of a TF family are likely to be responsible for binding *in vivo* to the hypomethylated enhancer probes identified above and regulating expression of their putative target genes, we analyzed the correlation between the probes containing a particular motif and expression of all known TFs (Fig. 6b, left). We ranked all the TFs by the degree to which their expression inversely correlated with the methylation status of the enhancers containing the motif (Fig. 6b, right), which allowed us to determine the family member most likely to be involved in regulation of the putative target genes in that particular cancer. For example, the GATA motif was enriched in (expression-linked) enhancer probes in BRCA samples (Fig. 6a). There are six members of the GATA family, with different members being linked to different differentiation phenotypes. For example, GATA1–3 have been linked to the specification of different hematopoietic cell fates and GATA4–6 are involved in differentiation of cardiac and lung tissues [44–49]. GATA3 is one of the most highly enriched transcription factors in the mammary epithelium, has been shown to be necessary for mammary cell differentiation, and is specifically required to maintain the luminal cell fate [48, 49]. Studies of human breast cancers have shown that GATA3 is expressed in early stage, well-differentiated tumors but not in advanced invasive cancers. In addition, GATA3 expression is correlated with longer disease-free survival and evidence suggests that it can prevent or reverse the epithelial to mesenchymal transition that is characteristic of cancer metastasis [50]. Not surprisingly, our analysis of the correlation of the methylation of the GATA motif-containing hypomethylated probes identified GATA3 as the most likely member of the GATA family to be responsible for the observed hypomethylation of GATA-containing enhancers in the BRCA samples (Fig. 6b). Not only was GATA3 the second most correlated transcription factor overall, but the extent of correlation made it easily distinguishable from other members (GATA3 had a *U* test *P* value less than 10^−40^, vs. *P* values greater than 10^−5^ for all other GATA family members). Furthermore, expression of GATA3 and methylation of GATA-containing enhancer probes were co-linked to breast cancer subtypes. As shown using color-coding in the Fig. 6b scatterplot, Luminal tumors had high expression of GATA3 and low methylation of GATA-containing enhancer probes, while Basal-like subtype tumors showed the converse. Figure 6c shows an example of one of these GATA-containing enhancer probes (cg1396202), along with the target gene (CCND1) predicted by expression to be regulated by this putative enhancer. ENCODE ChIP-seq data in the Luminal-subtype MCF7 cell line confirm that this putative enhancer region is indeed bound by GATA3, confirming the relationship between transcription factor binding and demethylation shown in [25]. This case was among the easier to detect, since breast cancer has two large subtypes (Luminal and Basal-like), which are molecularly quite distinct and are increasingly seen as two different diseases. As with all cancer genomic approaches, rarer subtypes will require larger number of samples to be identified by ELMER. Nevertheless, our results on other more challenging cancer types were also promising, as described below.

**Fig. 7:**
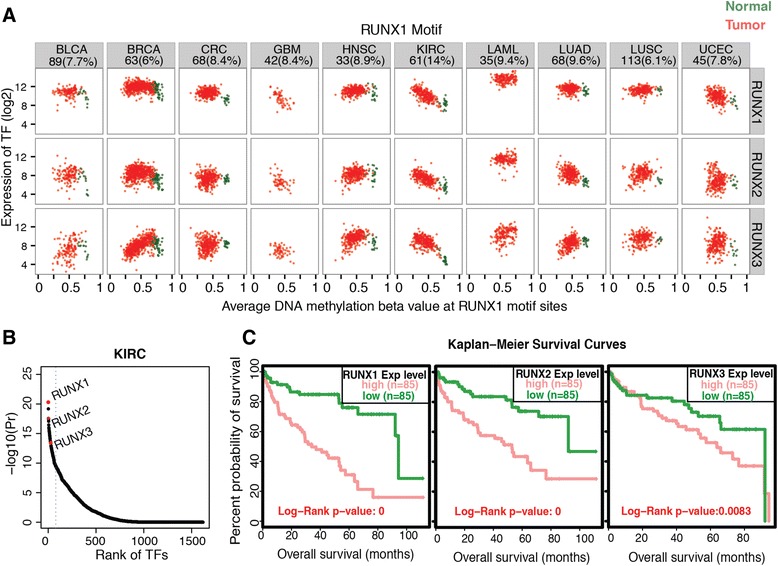
High RUNX1 expression is associated with poor survival in clear cell renal carcinoma. **a** Shown are scatter plots for the average DNA methylation at hypomethylated-paired probes containing a RUNX motif, plotted against expression for RUNX family members RUNX1, RUNX2, and RUNX3. The number (and percentage) of hypomethylated-paired probes having a RUNX motif in each cancer type is indicated underneath the name of each cancer type. **b** The ranked TF plot, as described in Fig. 6, is plotted for the RUNX motif in clear cell renal carcinoma (KIRC); RUNX1, RUNX2, and RUNX3 are all within the top 5 % (dotted line) of all TFs. (**c**) Kaplan-Meier survival curves for TCGA KIRC samples, stratified by expression of RUNX1 (left), RUNX2 (middle), or RUNX3 (right). In each plot, the survival data for patients having tumors with the highest (top 30 %) vs. lowest (bottom 30 %) expression for the given RUNX family member is shown; the Log-Rank test *P* value between the high and low groups is indicated

The same correlation analysis was performed for all motifs enriched in hypomethylated enhancer probes, and the most highly correlated member of the TF family expected to bind to each motif was identified (Additional files 9 and 10). In all, we identified 38 enhancer-TF pairs in the 10 tumor types. Although some of these TFs have previously been implicated in tumor development in the cancer type in which they were identified (for example, GATA3 in BRCA), many other associations were novel and provide new hypotheses regarding basic cancer biology and new potential targets for cancer prevention and treatment. In order to investigate potential clinical relevance of the new TF networks identified, we searched for cases where the TF found to be overexpressed in a subset of cases was also linked to patient survival. Our TF family member analysis showed that RUNX1, RUNX2, and RUNX3 were all within the top 5 % of TFs correlated with hypomethylation of RUNX-containing enhancer probes in clear cell renal carcinoma (KIRC) (Fig. 7)a, b. Of these, RUNX1 and RUNX2 were very highly correlated, with RUNX3 being only moderately so (Fig. 7)a, b. When we investigated patient survival in KIRC, RUNX1 and RUNX2 had highly significant associations with poor survival outcome after controlling for other co-variates, while RUNX3 was more marginal (Fig. 7c and Additional file 11A). These results suggest that the identification of specific TFs based on enhancer methylation analysis may lead to new insights into tumor classification and clinical outcomes (other identified TFs with association to survival are listed in Additional file 11B).

## Discussion

In our studies, we have used tumor-specific changes of the DNA methylation status within distal enhancer regions to provide insight into the mechanisms of gene expression, transcription factor networks, and tumor classification. We have shown that this can be a powerful approach for generating hypotheses about master regulators in cancer, and we propose that ELMER analysis be applied along with other hypothesis-generating approaches in high throughput cancer genomics. For the TCGA Pan-Cancer dataset, we provide to the community prioritized lists of putative enhancer-target gene pairs for future validation, and lists of site-specific transcription factors that should be further investigated for their role in the development and progression of specific tumor types.

**Fig. 8:**
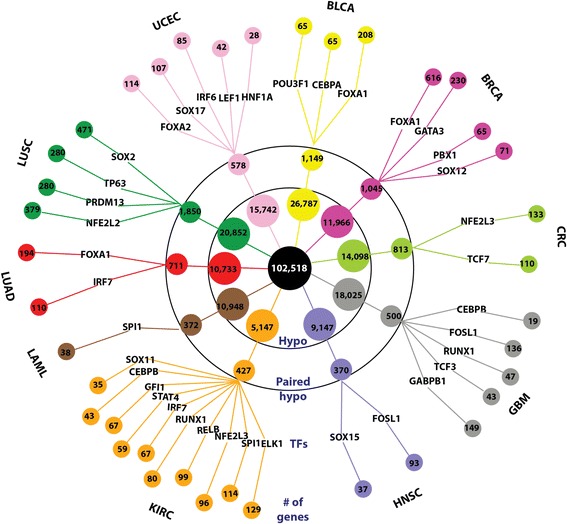
Identification of *in vivo* TF networks, including upstream TFs and downstream enhancers and gene targets. The innermost black circle represents the 102,518 distal enhancer probes from the HM450 platform. The next level (labeled Hypo) shows the number of hypomethylated distal enhancer probes identified in each cancer type. The third level (labeled Paired hypo) shows the number of hypomethylated probes that were significantly linked to a putative target gene in each cancer type. The number in the outermost level corresponds to the number of putative target genes (each linked by expression level to a specific hypomethylated enhancer) predicted to be regulated by the indicated TF (fourth level); where multiple TF family members were identified, only the most strongly associated family member is listed

Starting with a set of approximately 100,000 distal enhancer probes, we identified tens of thousands of enhancer regions that showed changes in methylation status in primary human tumors (Fig. 8). We identified many more hypomethylated (ostensibly activated) enhancers than hypermethylated (ostensibly deactivated) enhancers and have focused mainly on the hypomethylated enhancers in this study. We identified from 5,147 to 26,787 hypomethylated probes in different tumor types, corresponding to between 4,841 and 21,374 distinct enhancer regions. However, only a smaller subset of these hypomethylated enhancer probes (a total of 6,559 for all tumor types combined) could be linked to a putative target gene (based on expression levels of the 10 nearest genes upstream and 10 nearest genes downstream of the enhancer), ranging from a low of approximately 200 enhancer-putative target gene pairs in acute myelogenous leukemia to approximately 4,000 enhancer-putative gene pairs in lung cell squamous carcinomas. We feel that the expression filtering step is important for identifying those regions truly associated with enhancer-specific methylation, as other long-range methylation changes (such as global hypomethylation [14]) may also affect enhancer probes.

We found that most of the putative linkages between enhancer probes and local gene expression were cancer type-specific and that within each cancer type, most enhancers correlated with the expression of only one gene. In keeping with previous looping studies, we found that the putative target gene was typically not the nearest gene. In fact, the gene identified was the nearest gene in only approximately 15 % of the hypomethylated enhancer-gene pairs. As in other studies [51, 52], we found that the set of all hypomethylated enhancers was composed of similar proportions of intragenic and intergenic enhancers. We found that as compared to the intergenic enhancers, intragenic enhancers were 75 % more likely to be linked to expression of the nearest TSS (which in 88 % of the cases was the gene in which it resided); see Additional file 12. An intragenic enhancer can loop to regulate the ‘upstream’ promoter of the gene in which it resides but could also act as alternative promoter. Although we have eliminated all known promoters from our set of distal probes, we cannot eliminate the possibility that some of the intragenic enhancers represent as-of-yet unannotated, tumor-specific alternative promoters for the gene in which they reside [53, 54].

Our linking method is based strictly on correlation and therefore cannot absolutely rule out indirect (*trans*) interactions. For instance, if the same transcription factor or set of factors regulate both enhancer X and enhancer Y, the methylation patterns of X and Y across samples may be so similar that we link enhancer X to a gene that is in fact the direct target of enhancer Y. We have used high-confidence statistical thresholds in order to rule out as many of these indirect interactions as possible. Our search within the nearest 20 genes is unbiased, so the fact that we disproportionally find linkages to the gene nearest the enhancer probe provides strong evidence that we are identifying true direct (*cis*) interactions. We have provided a robust set of predicted linkages that can serve as a starting point for future experimental validations. Of course, we realize that we are working under a largely untested assumption that anti-correlation between an enhancer and expression level of a nearby gene indicate functional regulation. While this and prior correlative studies [22, 23, 25] provide strong supporting evidence for this, further experimental studies (for example, using CRISPR/Cas9 to delete the enhancers in appropriate tumor cell lines, followed by RNA-seq) will be needed to determine with certainty that the enhancers regulate their putative target genes, and what degree of correlation is required to infer functionality. Similarly, a comparison between our predicted enhancer-target pairs and global analysis of long-range chromatin looping would be of interest. Unfortunately, chromatin conformation assay data are not available for any of the tumor tissue samples and, in fact, very few studies of global chromatin looping have been completed for cancer cell lines. However, we have identified a set of chromatin loops derived from deep-sequenced ChIA-PET data from MCF7 cells [24]. Although MCF7 cells are not representative of all breast cancers (and are cultured cells, not tumor tissues), we did find that 166 of the 2,038 enhancer probes pairs we identified in breast cancer tumors (approximately 8%) were also identified as loops in the MCF7 ChIA-PET data. This was an almost four-fold enrichment over randomized enhancer probe-gene pairs (see Additional file 13 for an enrichment analysis, along with a complete list of BRCA enhancer-gene pairs falling within loops in MCF7 cells). We note that the various assays used to study looping are not yet optimized and do not always identify the same sets of loops [55]; in addition, some loops may not be related to transcriptional regulation. Thus, enhancer-gene pairs identified by expression assays are not necessarily concordant with the sets of promoter-enhancer loops identified by chromatin confirmation assays. Future comparisons between indirect (that is, correlative) mapping of enhancer-gene interactions of the type we described here, with direct physical mapping of enhancer-gene interactions, will be important to help to resolve the different mechanisms involved. However, in addition to the genome-wide confirmation by ChIA-PET, we note that at least two of the putative enhancer-gene pairs from our analysis have been studied in functional models confirming our results. The putative *CCND1* enhancer we identified in breast tumors (Fig. 6c) was shown to directly regulate the CCND1 gene in response to estradiol in breast cancer cells [56] and a putative MYC enhancer we identified in colon tumors (Additional file 14) was shown to be directly responsible for MYC expression in colon cancer cells [57], and *in vivo* in a mouse model of colorectal cancer [58].

We realize that the relationship between TF binding and DNA methylation can be complex [18]. For example, reduced DNA methylation in an enhancer region in a tumor cell relative to a normal cell could allow a TF to bind and regulate a target gene in a tumor-specific manner without changes in the expression level of that TF in the tumor. However, it is likely that increased levels of a TF in a tumor can result in higher binding at a partially methylated enhancer, directly leading to loss of DNA methylation [17]. Based on this second mechanism, we have attempted to identify TFs that regulate the target genes of enhancers that are hypomethylated in tumors. First, we identified a list of site-specific TF binding motifs that are enriched within the enhancers linked to putative target genes. Then, by examining the expression patterns of each of the TF family members expected to bind to these motifs, we have predicted the TF that regulates specific sets of genes in the different cancer types (Fig. 8). For example, in bladder cancer (BLCA) we have provided a list of 65, 208, and 65 genes that may be regulated by POU3F1, FOXA1, or CEBPA, respectively, by binding to a specific hypomethylated enhancer. In all, utilizing enhancer methylation patterns, expression of putative target genes, motif enrichment, and expression of TF family members that bind to the motif, we have derived a list of 4,280 enhancer-TF-putative target gene linkages.

Some of the cancer type-specific TF networks we show in Fig. 8 are already known to have a functional role in the same tumor type, such as PU.1 in AML [59] and TCF7L2 in colorectal cancer [28, 60–63]. Two of the four TFs we identified in squamous cell lung cancer (LUSC), TP63 and SOX2, are oncogenes that are overexpressed in LUSC through genomic amplification [64, 65]. Recently, SOX2 and TP63 were shown to interact functionally and co-localize to a large number of genomic binding sites in squamous cell lung cancer [66]. In a number of cases, incorporating TF expression data allowed us to resolve between different members of the same family that would be indistinguishable by binding motif alone. For instance, FOXA1 clearly appears to be responsible for hypomethylation of FOX-containing enhancers in breast (BRCA) and bladder (BLCA) cancers, while FOXA2 appears to be responsible in endometrial (UCEC). Other TF networks we identified, such as RUNX1/2 and its association with poor outcome in kidney cancer, have never been reported and will form the basis for future studies.

The method we describe herein is based on detecting methylation and expression differences between samples of the same tumor type, and is therefore aimed at identifying changes that co-occur within particular subsets of cases. For instance, we found that GATA-containing enhancer hypomethylation occurred primarily in the subset of breast cancer cases belonging to the Luminal subtype, which also had high expression of the GATA3 gene (Fig. 6b, c). While GATA3 is a well-studied case, our method can be applied to identify, understand, and find biomarkers for novel molecular subtypes. Understanding the genome-wide transcriptional consequences of molecular subtypes will be particularly relevant for those that are defined by genetic mutation of transcriptional regulators; indeed, transcription factors make up the largest functional class within the list of 127 cancer genes with so-called ‘driver’ mutations identified by TCGA [67]. A number of the altered transcription factor networks we identified using ELMER (Fig. 8) were also present within the 30 or so transcription factors included in this TCGA driver gene list. These TFs included FOXA1, FOXA2, GATA3, NFE2L2, and SOX17. Intriguingly, ELMER often identified a particular TF in the same cancer type or types where it is most frequently mutated. For instance, FOXA1 is most frequently mutated in Breast and Bladder cancer, and ELMER identified it in these specific cancers. Likewise, FOXA2 and SOX17 are primarily mutated in endometrial cancers, and ELMER identified network alterations specifically in this cancer type (UCEC). NFE2L2 is most frequently mutated in lung squamous cell carcinoma (LUSC), the same cancer type where ELMER detected NFE2L2 alterations. It will take additional work to understand the relationship between genetic mutations of TFs and epigenetic/transcriptomic changes in each of these different examples, but the identification of important cancer driver genes underscores the power of studying enhancers, which sit at the cis-regulatory interface between transcription factors, epigenetic modifiers, and downstream effector genes.

We also note that in some cases, transcription factors that are not expected to bind to the specific motif being analyzed were identified as being highly correlated with the degree of enhancer hypomethylation. In all, we identified 186 TFs frequently correlated with multiple motifs that do not correspond to the known motif for that TF family (Additional file 15). These correlations could be due to indirect effects caused by TF networks. For example, transcription factors regulated by GATA3 may show a similar correlation of expression with the hypomethylated probes in BRCA as does GATA3 itself. Another possible cause is suggested by the case of AP-1. Our results indicate that hypomethylation of AP-1-containing enhancers is a common feature of many or most cancer types (including nine of our 10 cancer types, see Fig. 6a); this confirms our earlier whole-genome observations in colorectal cancer [21]. While the AP1 motif is classically described as a binding sequence for FOS/JUN dimers, it is found to be enriched in many ChIP-seq datasets, including those using antibodies that recognize factors other than FOS or JUN family members [68]. Phosphorylation of JUN can lead to histone acetylation at AP-1 motif-containing enhancers by inhibiting their association with the Mbd3 component of the NuRD complex [69]. This could in turn allow binding of other positive transcriptional regulators, activation of downstream genes, and a proliferative expression program. Because JUN activity is regulated post-transcriptionally, it is logical that our method (which is based on expression) would miss JUN itself, and instead identify the positive regulators binding these regions (which are often cell-type specific). For instance, the most strongly associated TF with the AP-1 motif in kidney cancer is RUNX1, while in breast cancer it is FOXA1, suggesting that many of the AP-1 motif-containing sites may require AP-1 dependent de-repression along with positive RUNX1/FOXA activation.

Also included in the list of 186 ‘commonly correlated’ TFs are around 50 zinc finger domain-containing TFs (known as ZNFs). Although ZNFs are the most abundant class of human site-specific TFs, comprising around half of all site-specific TFs [70–72], few of them have been well studied. One of the commonly correlated factors was ZNF703, which correlated with 16 different motifs in the BRCA samples. Interestingly, high expression of ZNF703 has been shown to correlate with poor prognosis in patients with luminal B breast cancer [73]. We suggest that our analyses can point to a role for other ZNFs in tumorigenesis. In fact, 11 of the identified ZNFs showed associations with survival of the cancer in which they were identified (Additional file 16). For example, ZNF273 was correlated with four motifs in CRC and ZNF683 was correlated with nine motifs in KIRC; neither of these TFs has ever been associated with cancer. However, there is a strong correlation between high expression of ZNF273 and ZNF683 with poor survival rates in colorectal and kidney cancers, respectively. Most of the time, the 186 ‘commonly correlated’ TFs showed cancer type-specific correlations. However, one factor (GRHL2) was identified in the top 1 % of all correlations for 31 different motifs spread among five of the 10 different cancer types studied. GRHL2 has been shown to directly bind and activate the hTERT promoter and has been suggested to be involved in telomerase activation during cellular immortalization [74]. Perhaps GRHL2 plays an important role in tumor development in many cancer types.

The results we describe here use motif analysis primarily to help identify the transcription factors responsible for enhancer hypomethylation. However, the most important output of this work may actually be the identification of enhancers in which mutations in individual transcription factor binding sites can be responsible for cancer risk or cancer progression. A number of studies have shown that population risk alleles for cancer reside preferentially in enhancer regions [31, 75–79] and a recent paper demonstrated that these could be identified in breast cancer by combining DNA methylation and chromatin conformation capture data to identify putative enhancers [25]. Somatic enhancer mutations are predicted to affect cancer progression, although these have not yet been identified due to the overwhelming use of exon sequencing as a means to identify new cancer mutations. The recent availability of whole-genome sequencing of tumors has started to allow the identification of non-coding mutations, which have been shown to affect transcription factor binding sites [80–82]. Methods like ELMER, which can identify *in vivo* enhancer regions in tumors, will be essential for analyzing non-coding cancer mutations arising from WGS studies.

## Conclusions

Although our study is not comprehensive due to the nature of the DNA methylation platform used by TCGA (which only contains coverage of 15 % of known enhancers) and because enhancers have not yet been mapped in all normal and tumor cell types, our analyses have allowed us to identify a number of cancer type-specific transcriptional regulators, along with the *cis*-regulatory sequences mediating effects on target genes. Large-scale identification of such *cis*-regulatory regions will be critical for understanding the effects of non-coding genetic polymorphisms on cancer risk and non-coding somatic mutations on cancer progression [28, 59, 60]. Complete tumor methylation profiles using whole-genome bisulfite sequencing [21, 23, 83] are rapidly becoming available, and these will dramatically increase the power of the ELMER approach to reconstruct complete transcription factor network and identify important cis-regulatory regions.

## Methods

### Availability of source code and R package

All source code is available as an R package, ELMER, downloadable from the main Bioconductor repository [84] or from our GitHub repository [85]. Vignettes illustrating the use of the functions are available as part of the BioConductor package, along with an example replicating the results described in this paper using the ELMER function TCGA.pipe. A user manual and tutorial can be downloaded from the GitHub repository here: [86], and a full manual can be downloaded here: [87].

### DNA methylation and RNA-seq datasets

TCGA level 3 DNA methylation data based on the Illumina Infinium HumanMethylation450 BeadArray platform was downloaded from [88]. Only the samples whitelisted by TCGA for Pan-Cancer Analysis Working Group were used in the study. The whitelist can be downloaded from Sage Bionetworks Synapse [89] with identifier syn1571603. The version numbers and final sample IDs for each cancer type are listed in Additional file 1. The DNA methylation level at each CpG is referred to as a beta (β) value, calculated as (M/(M+U)), where M represents the methylated allele intensity and U the unmethylated allele intensity, which are normalized using the TCGA standard pipeline. Beta values are in the range of 0 to 1, reflecting the fraction of methylated alleles at each CpG in the each tumor; beta values close to 0 indicating low levels of DNA methylation and beta values close to 1 indicating high levels of DNA methylation. Since there are no available normal tissues for acute myeloid leukemia (LAML) and glioblastoma multiforme (GBM) in TCGA, we also downloaded Infinium HM450K DNA methylation data from publicly available sources as normal tissue controls for these two cancer types. A set of 58 sorted glial cell samples from GEO (accession number GSE41826) was used as normal reference samples for glioblastoma. A set of 11 sorted blood samples from GEO (accession number GSE49618) was used for normal reference samples for leukemia. These data were generated at the USC Epigenome Center and were processed through the same data analysis pipeline that was used to create the TCGA Level 3 data files (all TCGA data were also generated by the USC Epigenome Center). The sample IDs are also listed in Additional file 1.

TCGA Level 3 RNA-seq data were downloaded from [88]. The version number of each package downloaded is listed in Additional file 1. TCGA uses gene-level expression values, meaning any alternative isoforms are included in a single normalized RSEM expression value. TCGA data production and analysis pipelines are described elsewhere, but a brief description follows: all data were generated on the Illumina HiSeq platform, with the exception of UCEC, which was generated on the Illumina GAII platform. Within each cancer type, data were mapped with MapSplice and quantitated with RSEM (RNA-seq by Expectation Maximization). RSEM outputs expression values that are normalized across samples, so that the third quartile for each sample equals 1,000. Entrez gene IDs were used for mapping to genomic locations using GenomicRanges [90]. The final RNA-seq sample IDs used in our analyses are listed in Additional file 1.

### Selecting enhancer probes

Probes overlapping SNPs are removed as part of the standard TCGA Level 3 pipeline. Probes located less than 2 kb from an annotated transcription start site in GENCODE v.15 were filtered out to remove promoter regions from our analysis. ENCODE/REMC chromHMM data were downloaded from [91] and any HM450 probes falling within the genomic regions annotated as EnhG1, EnhG2, EnhA1, or EnhA2 were selected. FANTOM5 data were downloaded from [92] and any HM450 probes falling within regions annotated as eRNA were selected. This resulted in 102,518 enhancer probes, which are listed in Additional file 2. This functionality is implemented in the get.feature.probe function of the ELMER BioConductor package.

### Identifying enhancer probes with cancer-specific DNA methylation changes

Each of the 10 cancer types was processed independently to identify cancer-specific DNA methylation changes. For each enhancer probe, we first ranked tumor samples and normal samples (within the cancer type) by their DNA methylation beta values. To identify hypomethylated probes, we compared the lower normal quintile (20 % of normal samples with the lowest methylation) to the lower tumor quintile (20 % of tumor samples with the lowest methylation), using an unpaired one-tailed *t*-test. Only the lower quintiles were used because we did not expect all cases to be from a single molecular subtype, and we sought to identify methylation changes within cases from the same molecular subtype. Twenty percent (that is, a quintile) was picked as a cutoff to include high enough sample numbers to yield *t*-test *P* values that could overcome multiple hypothesis correction, yet low enough to be able to capture changes in individual molecular subtypes occurring in 20 % or more of the cases. This number can be set arbitrarily as an input to the get.diff.meth function in the ELMER package, and should be tuned based on sample sizes in individual studies. The one tailed *t*-test was used to rule out the null hypothesis: μ_tumor_ ≥ μ_normal_, where μ_tumor_ is the mean methylation within the lowest tumor quintile and μ_normal_ is the mean within the lowest normal quintile. Raw *P* values were adjusted for multiple hypothesis testing using the Benjamini-Hochberg method, and probes were selected when they had adjusted *P* value less than 0.01. For additional stringency, probes were only selected if the methylation difference |Δ|= |μ_normal_ - μ_tumor_ | was greater than 0.3. This technique is illustrated in Fig. 1b, and carried out in the get.diff.meth function of the ELMER package. The same method was used to identify hypermethylated probes, except we used upper tumor quintile and upper normal quintile, and chose the opposite tail in the *t*-test. The full set of hypermethylated and hypomethylated probes we identified are provided in Additional file 3, and can be replicated using the TCGA.pipe vignette in the ELMER package.

### Linking enhancer probes with methylation changes to target genes with expression changes

For additional stringency and to avoid correlations due to non-cancer contamination, we selected only those enhancer probes that had differential methylation as defined above, and where at least 5 % of all samples (combining tumor and normal) had beta values >0.3. Then, for each of these differentially methylated enhancer probes, the closest 10 upstream genes and the closest 10 downstream genes were tested for correlation between methylation of the probe and expression of the gene. To select these genes, the probe-gene distance was defined as the distance from the probe to a transcription start site specified by the TCGA RNA-seq Level 3 data files. We used the Level 3 TCGA RNA-seq data files; these represent expression at the gene level, and merge any alternate transcript isoforms into a single expression value for each gene. Thus, exactly 20 statistical tests were performed for each probe, as follows. For each probe-gene pair, the samples (all tumors and normals within a particular cancer type) were divided into two groups: the *M* group, which consisted of the upper methylation quintile (the 20 % of samples with the highest methylation at the enhancer probe), and the *U* group, which consisted of the lowest methylation quintile (the 20 % of samples with the lowest methylation.) The 20 % cutoff is a configurable parameter in the get.pair function of ELMER. We used 20 % as a balance, which would allow us to identify changes in a molecular subtype making up a minority (that is, 20 %) of cases, while also yielding enough statistical power to make strong predictions. For each candidate probe-gene pair, the Mann-Whitney *U* test was used to test the null hypothesis that overall gene expression in group *M* was greater or equal than that in group *U*. This non-parametric test was used in order to minimize the effects of expression outliers, which can occur across a very wide dynamic range. For each probe-gene pair tested, the raw *P* value *P*_*r*_ was corrected for multiple hypothesis using a permutation approach as follows (implemented in the get.permu function of the ELMER package). The gene in the pair was held constant, and 10,000 random methylation probes were used to perform the same one-tailed *U* test, generating a set of 10,000 permutation *P* values (*P*_*p*_). We chose the 10,000 random probes only from among those that were ‘distal’ (greater than 2 kb from an annotated transcription start site), in order to make these null-model probes qualitatively similar to the probe being tested. We only used non-enhancer probes, as using enhancer probes would introduce large numbers of co-regulated enhancers. An empirical *P* value *P*_*e*_ value was calculated using the following formula (which introduces a pseudo-count of 1):

$$ Pe=\frac{num\left(Pp\le Pr\right)\kern0.5em +\kern0.5em 1}{10001} $$

### ChIA-PET analysis

MCF7 ChIA-PET linkage pairs were taken from a previous publication [24]. The random pairs were generated by randomly selecting the same number of probes from the set of distal enhancer probes, and pairing each with one or more of the 20 adjacent genes; the number of links made for each random probe was identical to the corresponding ‘true’ probe. Thus, the random linkage set has both the same number of probes and the same number of linked genes as the true set. One hundred such random datasets were generated to arrive at a 95 % CI (+/-1.96* SD).

### Gene Ontology (GO) enrichment analysis

Genes associated with hypo- or hypermethylated enhancer probes in more than one cancer type were selected for GO analysis. GO analyses were performed using the R package ‘topGO’ [93]. The classic Fisher test was used to generate enrichment *P* values. To select the GO terms that pass a significance cutoff, *P* values were adjusted using the Benjamini-Hochberg method; only those GO terms with a *P* value <0.01 and a fold change >1.5 are shown in Fig. 5.

### Motif analyses

We used FIMO [94] with a *P* value <1e–4 to scan a +/- 100 bp region around each probe using Factorbook motif position weight matrices (PWMs) [39, 95] and Jasper core human motif PWMs generated from the R package MotifDb [96]. For each probe set tested (that is, the list of gene-linked hypomethylated probes in a given cancer type), a motif enrichment OR and a 95 % CI were calculated using following formulas:

$$ \begin{array}{l}p=\frac{a}{\left(a+b\right)}\\ {}P=\frac{c}{\left(c+d\right)}\\ {} Odds\  Ratio = \frac{p/\left(1-p\right)}{P/\left(1-P\right)}\\ {}SD=\sqrt{\frac{1}{a}+\frac{1}{b}+\frac{1}{c}+\frac{1}{d}}\\ {} lower\  boundary\  of\ 95\%\  confidence\  interval= \exp \left( \ln (OR)-SD\right)\end{array} $$

where *a* is the number of probes within the selected probe set that contain one or more motif occurrences; *b* is the number of probes within the selected probe set that do not contain a motif occurrence; *c* and *d* are the same counts within the entire enhancer probe set. A probe set was considered significantly enriched for a particular motif if the 95 % CI of the OR was greater than 1.1, and the motif occurred at least 10 times in the probe set. As described in the text, ORs were also used for ranking candidate motifs. This analysis is implemented in the get.enrichmed.motifs function of the ELMER package.

### Associating TF expression with TF binding motif methylation

For each motif considered to be enriched within a particular probe set, we compared the average DNA methylation at all distal enhancer probes within +/− 100bp of a motif occurrence, to the expression of 1,777 human TFs ([97] and with further refinements, see Additional file 17). A statistical test was performed for each motif-TF pair, as follows. The samples (all tumors and normal within a particular cancer type) were divided into two groups: the *M* group, which consisted of the 20 % of samples with the highest average methylation at all motif-adjacent probes, and the *U* group, which consisted of the 20 % of samples with the lowest methylation. The 20th percentile cutoff is a parameter to the get.TFs function of the ELMER package, and was set to allow for identification of molecular subtypes present in 20 % of cases. For each candidate motif-TF pair, the Mann-Whitney *U* test was used to test the null hypothesis that overall gene expression in group *M* was greater or equal than that in group *U*. This non-parametric test was used in order to minimize the effects of expression outliers, which can occur across a very wide dynamic range. For each motif tested, this resulted in a raw *P* value (*P*_*r*_) for each of the 1,777 TFs. All TFs were ranked by the -log10(*P*_*r*_), and those falling within the top 5 % of this ranking were considered candidate upstream regulators. The best upstream TFs for each of these cases was automatically extracted as high-value candidates, and presented in Fig. 8. These high-value candidates are also shown in detail in Additional files 9 and 10.

### Survival analyses

A Kaplan-Meier survival analysis was used to estimate the association of the TF expression with the survival of patients. For each selected TF and cancer type combination, tumor samples with the highest (top 30 %) and lowest (bottom 30 %) transcription factor expression were analyzed using a Log Rank test. Overall survival was calculated from the date of initial diagnosis of cancer to disease-specific death (patients whose vital status is termed dead) and months to last follow-up (for patients who are alive).

### Data access

The TCGA samples can be downloaded at https://tcga-data.nci.nih.gov/tcgafiles/ftp_auth/distro_ftpusers/anonymous/tumor/. The whitelist from Pan-Can group is available on Synapse (https://www.synapse.org/) as syn1571603. The enhancer genomic coordinates can be downloaded at http://egg2.wustl.edu/roadmap/data/byFileType/chromhmmSegmentations/ChmmModels/coreMarks/jointModel/final/) and http://enhancer.binf.ku.dk/Welcome.html.

## Additional files

Additional file 1:
**TCGA DNA methylation and RNA-seq sample ID numbers.** The data platform and archive version number are listed in the sheet named ‘Version number’. The ‘DNA methylation sample ID’ sheet provides information concerning the TCGA sample ID, the tissue type (normal or tumor), and the cancer type for the DNA methylation datasets. The ‘RNA-seq sample ID’ sheet provides information concerning the TCGA sample ID, the tissue type (normal or tumor), and the cancer type for the RNA-seq datasets. Additional file 2:
**Distal enhancer probes on the HM450 array.** The chromosomal location and the name of each of the 102,518 distal enhancer probes used in this study are indicated. Additional file 3:
**Hypo- and hypermethylated enhancer probes identified for each tumor type.** Individual worksheets are provided that list the hypermethylated probes and hypomethylated probes identified for each specific cancer type. Additional file 4:
**Probe-gene pairs showing inverse correlations between methylation and expression.** Individual worksheets are provided that list all of the significant probe-gene pairs for hypomethylated probes and hypermethylated probes in each cancer type. Pr represents real *P* value from the Mann-Whitney *U* test for each pair; Pe represents the empirical *P* value for each pair; the distance between the probes and the putative target genes are shown in the Distance column; the ranking based on the relative distance of the putative target gene among the 20 adjacent genes (10 on either side of the enhancer) is shown; and the cancer type (CT) is indicated. The *P* value for promoter methylation’ column specifies the anti correlation between methylation at the promoter itself and the expression level of the gene. This is calculated using the same Mann-Whitney statistic we use to evaluate enhancer-expression correlation, but we average beta values within the standard promoter methylation region, from -300 to +500 bp relative to the transcription start site (TSS). This region is consistently methylated in all active promoters based on whole-genome bisulfite sequencing (in cell lines [95] and primary TCGA tumors, manuscript in preparation). As with our enhancer method, we select for strong changes in methylation filtering out any case where 95 % of samples have methylation less than 0.3; in the spreadsheet, these have a *P* value of 1.0. The worksheet ‘tumor vs. normal expression’ contains a table showing that the majority of target genes linked to hypermethylated enhancers have lower expression in tumors than normal tissues, while the majority linked to hypomethylated enhancers have higher expression in tumors. The worksheet ‘promoter methylation’ shows the fraction of enhancer-linked genes in each cancer type that also have significant correlation with methylation of the promoter. It is under 10 % of genes for all cancer types. Additional file 5:
**Quantitative summary of links, probes, and genes for each cancer type.** (**A**) Shown are histograms representing the number of putative probes-gene pairs, the number of total probes in the set of paired-probes, and the number of total genes in the set of paired probes for the set of hypomethylated (top) and hypermethylated (bottom) probe-gene pairs in each cancer type. For each plot, the number of probes identified in one or more tumor types is indicated by the colored bars. (**B**) Shown is a heatmap illustrating the similarity of probe-gene pairs, probes in the pairs, and genes in the pairs among the different cancer types. The color bar indicates the OR for the similarity (overlap) between the indicated cancer types (a higher OR indicates a more significant similarity). Additional file 6:
**Rank of putative target gene according to distance in the enhancer-gene pairs for each cancer type.** (**A**) Shown is the distribution for the ranking (by distance) of each putative target gene linked to an enhancer for enhancers that are significantly associated with more than one gene. (**B**) Shown is the distribution for the ranking (by distance) of each putative target gene linked to an enhancer for each cancer type. The left panel shows the pairs for which the enhancer is significantly associated with more than one gene and the right panel shows the pairs for which the enhancer is significantly associated with only one gene. Additional file 7:
**Summary of enriched motifs for enhancer-gene pairs with hypomethylated distal enhancers.** On the worksheet entitled ‘Summary’, the fold enrichment of each indicated motif in a specific cancer type (CT) is shown. Shown in the Enhancer column is the number of the paired enhancers (after clustering distal enhancer probes within 500 bp) containing the enriched motif (the percentage of total paired hypomethylated enhancers in that cancer type containing each motif is shown in parentheses). For each motif, also shown is the number of genes linked to the probes containing the motif and the number of probe-gene pairs. The worksheet entitled ‘Detail’ contains information for each individual probe linked to a putative target gene via a distal region containing an enriched motif. Pe represents the empirical *P* value for each pair; the distance between the probes and the putative target genes are shown in the Distance column; the ranking based on the relative distance of the putative target gene among the 20 adjacent genes (10 on either side of the enhancer) is shown; and the cancer type (CT) is indicated. Additional file 8:
**Motif enrichment heatmaps.** (**A**) Shown are the heatmaps for motifs that are enriched in the sets of all hypomethylated probes (top panel) and all hypermethylated probes (bottom panel). (**B**) Shown are the heatmaps for motifs that are enriched in the sets of only those hypomethylated (top panel) or hypermethylated (bottom panel) probes that are linked to putative target genes (B bottom panel). Additional file 9:
**Plots of association between all human TFs and DNA methylation at enriched motif sites.** Shown are TF ranking plots based on the score (-log10(Pr)) of association between TF expression and DNA methylation of the motif in the cancer type in which the motifs are enriched. The dashed blue line indicates the boundary of the top 5 % association score. The top three associated TFs and the TF family members (dots in red) that are associated with that specific motif are labeled in the plot. Additional file 10:
**Scatter plots for TF family members significantly associated with DNA methylation at distal enhancer regions having enriched motifs.** Shown are scatter plots for average DNA methylation at probes having the indicated enriched motif (x axis, shown on the top of each set of panels) vs. the expression of the significantly correlated motif-relevant TF family members (y axis, shown on the right side of each panel). Each dot represents a different patient sample; red and green indicate the tumor and normal samples, respectively. Pairs that are within the top 5 % of TFs linked to a given motif are indicated with a number inside the cell. The number corresponds to the rank of the given TF relative to all 1,777 TFs (with ‘1’ being the most strongly correlated). Additional file 11:
**Survival plots for TF family members significantly associated with DNA methylation at distal enhancer regions having enriched motifs.** (**A**) The output of a Cox model regression analysis for the effects of expression of RUNX1 on survival within KIRC samples. Leukocyte methylation signature was calculated as in (PMID 22120008), and staging information was taken from TCGA clinical data. Leukocyte methylation signature was included to rule out RUNX1 expression from contaminating leukocytes, which are the main source of non-cancer cells in KIRC samples. (**B**) Kaplan-Meier survival curves for TF family members significantly associated with DNA methylation at the distal enhancer regions with enriched motifs in the indicated cancer type. The survival data for patients having tumors with the highest (top 30%) and lowest (bottom 30%) transcription factor expression are shown; the Log Rank test *P* value between the high and low groups is indicated. Additional file 12:
**Proportion of intragenic vs. intergenic enhancers that regulate the nearest gene.** Shown are bar graphs indicating the number of intergenic vs. intragenic enhancers, the number of each category that is associated with expression of the nearest gene, and the number of intragenic enhancers associated with expression of the nearest gene with that gene being the one in which the enhancer resides. Additional file 13:
**Overlapping analysis between putative probe-gene pairs in BRCA and interactions from ChIA-PET data from the MCF7 breast cancer cell line.** A list of putative probe-gene pairs in BRCA that overlap with interactions from ChIA-PET data from the MCF7 cell line is provided. The bar graph shows the comparison of the number of probe-gene pairs identified within MCF7 ChIA-PET data using the putative pairs from BRCA vs. random pairs. The random pairs were generated by randomly selecting the same number of probes from the set of distal enhancer probes, and pairing each with one or more of the 20 adjacent genes; the number of links made for each random probe was identical to the corresponding ‘true’ probe. Thus, the random linkage set has both the same number of probes and the same number of linked genes as the true set. One hundred such random datasets were generated to arrive at a 95 % CI (+/-1.96* SD). Additional file 14:
***MYC*** **3’ end enhancer regulates** ***MYC*** **expression in colorectal cancer tissue.** (**A**) Shown is a scatter plot showing DNA methylation at probes located at the 3’ end of the *MYC* gene vs. the expression of *MYC* RNA. Each dot represents a different patient sample; red and green indicate the tumor and normal samples, respectively. (**B**) Shown is the location of the *MYC* 3’ enhancer and the ENCODE ChIP-seq histone and transcription factor tracks from the University of California, Santa Cruz genome browser. The green bar indicates the location of enhancer that has been previously identified to regulate *MYC* expression in the HCT116 colon cancer cell line [57, 58]. Additional file 15:
**Transcription factors significantly associated with multiple different motifs.** Each row represents individual transcription factors and each column represent different cancer types. The numbers in the table show the number of enriched motifs that the transcription factors associate with in each cancer type; the transcription factor must be in the top 1 % of all ranked TFs for that specific motif in that specific cancer type to be listed on the table. Additional file 16:
**Survival analysis of commonly identified ZNFs.** (**A**) Shown is a table listing the subset of ZNFs (the entire list can be found in Additional file 17) which were identified in the top 1 % of ranked TFs, which were significantly associated with multiple different motifs in a specific cancer type (the number of motifs with which the TF was associated is listed in parentheses), and whose expression level significantly correlates with patient survival. The direction of correlation is labeled in column labeled ‘Survival’ (red and green color represents high expression correlated with worse survival or better bad survival, respectively) and log Rank test *P* value between the high and low expression groups is provided in the column labeled ‘logRankP’. (**B**) Shown are example Kaplan-Meier survival curves for two ZNFs. The survival data for patients having tumors with the highest (top 30 %) and lowest (bottom 30 %) transcription factor expression is shown; the Log Rank test *P* value between the high and low groups is indicated. Additional file 17:
**List of the TFs used in this study.** Shown is the list of TFs used to compare expression analysis of all TFs to motif methylation in the different cancer types, taken from [97].

## References

[1] Bernstein BE, Birney E, Dunham I, Green ED, Gunter C, Snyder M (2012). An integrated encyclopedia of DNA elements in the human genome. Nature..

[2] Roadmap Epigenomics Consortium (2015). Integrative analysis of 111 reference human epigenomes. Nature.

[3] Henikoff S (2007). ENCODE and our very busy genome. Nat Genet..

[4] Filion GJ, Van Bemmel JG, Braunschweig U, Talhout W, Kind J, Ward LD (2010). Systematic protein location mapping reveals five principal chromatin types in Drosophila cells. Cell..

[5] Ernst J, Kheradpour P, Mikkelsen TS, Shoresh N, Ward LD, Epstein CB (2011). Mapping and analysis of chromatin state dynamics in nine human cell types. Nature..

[6] Hoffman MM, Buske OJ, Wang J, Weng Z, Bilmes J, Noble WS (2012). Unsupervised pattern discovery in human chromatin structure through genomic segmentation. Nat Methods..

[7] Creyghton MP, Cheng AW, Welstead GG, Kooistra T, Carey BW, Steine EJ (2010). Histone H3K27ac separates active from poised enhancers and predicts developmental state. Proc Natl Acad Sci U S A..

[8] Rada-Iglesias A, Bajpai R, Swigut T, Brugmann SA, Flynn RA, Wysocka J (2011). A unique chromatin signature uncovers early developmental enhancers in humans. Nature..

[9] Bernstein BE, Stamatoyannopoulos JA, Costello JF, Ren B, Milosavljevic A, Meissner A (2010). The NIH Roadmap Epigenomics Mapping Consortium. Nat Biotechnol..

[10] Beck S, Bernstein BE, Campbell RM, Costello JF, Dhanak D, Ecker JR (2012). A blueprint for an international cancer epigenome consortium. A report from the AACR Cancer Epigenome Task Force. Cancer Res.

[11] He B, Chen C, Teng L, Tan K (2014). Global view of enhancer-promoter interactome in human cells. Proc Natl Acad Sci U S A..

[12] Sheffield NC, Thurman RE, Song L, Safi A, Stamatoyannopoulos JA, Lenhard B (2013). Patterns of regulatory activity across diverse human cell types predict tissue identity, transcription factor binding, and long-range interactions. Genome Res..

[13] Pastor WA, Stroud H, Nee K, Liu W, Pezic D, Manakov S (2014). MORC1 represses transposable elements in the mouse male germline. Nat Commun..

[14] Bergman Y, Cedar H (2013). DNA methylation dynamics in health and disease. Nat Struct Mol Biol..

[15] Thomassin H, Flavin M, Espinás ML, Grange T (2001). Glucocorticoid-induced DNA demethylation and gene memory during development. EMBO J..

[16] Lister R, Pelizzola M, Dowen RH, Hawkins RD, Hon G, Tonti-Filippini J (2009). Human DNA methylomes at base resolution show widespread epigenomic differences. Nature..

[17] Stadler MB, Murr R, Burger L, Ivanek R, Lienert F, Schöler A (2011). DNA-binding factors shape the mouse methylome at distal regulatory regions. Nature..

[18] Blattler A, Farnham PJ (2013). Cross-talk between site-specific transcription factors and DNA methylation states. J Biol Chem..

[19] Ooi L, Wood IC (2007). Chromatin crosstalk in development and disease: lessons from REST. Nat Rev Genet..

[20] Gebhard C, Benner C, Ehrich M, Schwarzfischer L, Schilling E, Klug M (2010). General transcription factor binding at CpG islands in normal cells correlates with resistance to de novo DNA methylation in cancer cells. Cancer Res..

[21] Berman BP, Weisenberger DJ, Aman JF, Hinoue T, Ramjan Z, Liu Y (2012). Regions of focal DNA hypermethylation and long-range hypomethylation in colorectal cancer coincide with nuclear lamina-associated domains. Nat Genet..

[22] Aran D, Sabato S, Hellman A (2013). DNA methylation of distal regulatory sites characterizes dysregulation of cancer genes. Genome Biol..

[23] Hovestadt V, Jones DT, Picelli S, Wang W, Kool M, Northcott PA (2014). Decoding the regulatory landscape of medulloblastoma using DNA methylation sequencing. Nature..

[24] Li G, Ruan X, Auerbach RK, Sandhu KS, Zheng M, Wang P (2012). Extensive promoter-centered chromatin interactions provide a topological basis for transcription regulation. Cell..

[25] Aran D, Hellman A (2013). DNA methylation of transcriptional enhancers and cancer predisposition. Cell..

[26] Wiench M, John S, Baek S, Johnson TA, Sung MH, Escobar T (2011). DNA methylation status predicts cell type-specific enhancer activity. EMBO J..

[27] Weinstein JN, Collisson EA, Mills GB, Shaw KR, Ozenberger BA, Ellrott K (2013). The Cancer Genome Atlas Pan-Cancer analysis project. Nat Genet..

[28] The Cancer Genome Atlas (2012). Comprehensive molecular characterization of human colon and rectal cancer. Nature.

[29] GENCODE gene annotations. [http://www.gencodegenes.org/releases/15.html]

[30] Ernst J, Kellis M (2012). ChromHMM: automating chromatin-state discovery and characterization. Nat Methods..

[31] ENCODE_Project_Consortium (2012). An integrated encyclopedia of DNA elements in the human genome. Nature.

[32] Andersson R, Gebhard C, Miguel-Escalada I, Hoof I, Bornholdt J, Boyd M (2014). An atlas of active enhancers across human cell types and tissues. Nature..

[33] Kwasnieski JC, Fiore C, Chaudhari HG, Cohen BA (2014). High-throughput functional testing of ENCODE segmentation predictions. Genome Res..

[34] Blow MJ, McCulley DJ, Li Z, Zhang T, Akiyama JA, Holt A (2010). ChIP-Seq identification of weakly conserved heart enhancers. Nat Genet..

[35] Sanyal A, Lajoie BR, Jain G, Dekker J (2012). The long-range interaction landscape of gene promoters. Nature..

[36] Jin F, Li Y, Dixon JR, Selvaraj S, Ye Z, Lee AY (2013). A high-resolution map of the three-dimensional chromatin interactome in human cells. Nature..

[37] Rao SS, Huntley MH, Durand NC, Stamenova EK, Bochkov ID, Robinson JT (2014). A 3D map of the human genome at kilobase resolution reveals principles of chromatin looping. Cell..

[38] Mathelier A, Zhao X, Zhang AW, Parcy F, Worsley-Hunt R, Arenillas DJ (2014). JASPAR 2014: an extensively expanded and updated open-access database of transcription factor binding profiles. Nucleic Acids Res..

[39] Wang J, Zhuang J, Iyer S, Lin XY, Greven MC, Kim BH (2013). Factorbook.org: a Wiki-based database for transcription factor-binding data generated by the ENCODE consortium. Nucleic Acids Res..

[40] Fujiwara T, O'Geen H, Keles S, Blahnik K, Linnemann AK, Kang YA (2009). Discovering hematopoietic mechanisms through genome-wide analysis of GATA factor chromatin occupancy. Mol Cell..

[41] Xu X, Bieda M, Jin VX, Rabinovich A, Oberley MJ, Green R (2007). A comprehensive ChIP-chip analysis of E2F1, E2F4, and E2F6 in normal and tumor cells reveals iterchangeable roles of E2F family members. Genome Res..

[42] Shakya A, Callister C, Goren A, Yosef N, Garg N, Khoddami V (2015). Pluripotency transcription factor oct4 mediates stepwise nucleosome demethylation and depletion. Mol Cell Biol..

[43] Lee DS, Shin JY, Tonge PD, Puri MC, Lee S, Park H (2014). An epigenomic roadmap to induced pluripotency reveals DNA methylation as a reprogramming modulator. Nat Commun..

[44] Bresnick EH, Lee HY, Fujiwara T, Johnson KD, Keles S (2010). GATA switches as developmental drivers. J Biol Chem..

[45] Brewer A, Pizzey J (2006). GATA factors in vertebrate heart development and disease. Expert Rev Mol Med..

[46] Chou J, Provot S, Werb Z (2010). GATA3 in development and cancer differentiation: cells GATA have it!. J Cell Physiol..

[47] Patient RK, McGhee JD (2002). The GATA family (vertebrates and invertebrates). Curr Opin Genet Dev..

[48] Kouros-Mehr H, Bechis SK, Slorach EM, Littlepage LE, Egeblad M, Ewald AJ (2008). GATA-3 links tumor differentiation and dissemination in a luminal breast cancer model. Cancer Cell..

[49] Kouros-Mehr H, Kim JW, Bechis SK, Werb Z (2008). GATA-3 and the regulation of the mammary luminal cell fate. Curr Opin Cell Biol..

[50] Yan W, Cao QJ, Arenas RB, Bentley B, Shao R (2010). GATA3 inhibits breast cancer metastasis through the reversal of epithelial-mesenchymal transition. J Biol Chem..

[51] Heintzman ND, Stuart RK, Hon G, Fu Y, Ching CW, Hawkins RD (2007). Distinct predictive chromatin signatures of transcriptional promoters and enhancers in the human genome. Nature Genetics..

[52] Blattler A, Yao L, Witt H, Guo Y, Nicolet CM, Berman BP (2014). Global loss of DNA methylation uncovers intronic enhancers in genes showing expression changes. Genome Biol..

[53] Maunakea AK, Nagarajan RP, Bilenky M, Ballinger TJ, D’Souza C, Fouse SD (2010). Conserved role of intragenic DNA methylation in regulating alternative promoters. Nature..

[54] Kowalczyk MS, Hughes JR, Garrick D, Lynch MD, Sharpe JA, Sloane-Stanley JA (2012). Intragenic enhancers act as alternative promoters. Mol Cell..

[55] Raviram R, Rocha PP, Bonneau R, Skok JA (2014). Interpreting 4C-Seq data: how far can we go?. Epigenomics..

[56] Eeckhoute J, Carroll JS, Geistlinger TR, Torres-Arzayus MI, Brown M (2015). A cell-type-specific transcriptional network required for estrogen regulation of cyclin D1 and cell cycle progression in breast cancer. Genes & Dev..

[57] Yochum GS, Cleland R, Goodman RH (2008). A genome-wide screen for beta-catenin binding sites identifies a downstream enhancer element that controls c-Myc gene expression. Mol Cell Biol..

[58] Konsavage WM, Yochum GS (2014). The myc 3’ wnt-responsive element suppresses colonic tumorigenesis. Mol Cell Biol..

[59] Rosenbauer F, Wagner K, Kutok JL, Iwasaki H, Le Beau MM, Okuno Y (2004). Acute myeloid leukemia induced by graded reduction of a lineage-specific transcription factor, PU.1. Nat Genet.

[60] Bass AJ, Lawrence MS, Brace LE, Ramos AH, Drier Y, Cibulskis K (2011). Genomic sequencing of colorectal adenocarcinomas identifies a recurrent VTI1A-TCF7L2 fusion. Nat Genet..

[61] Frietze S, Wang R, Yao L, Tak YG, Ye Z, Gaddis M (2012). Cell type-specific binding patterns reveal that TCF7L2 can be tethered to the genome by association with GATA3. Genome Biol..

[62] Sur IK, Hallikas O, Vaharautio A, Yan J, Turunen M, Enge M (2012). Mice lacking a Myc enhancer that includes human SNP rs6983267 are resistant to intestinal tumors. Science..

[63] Pomerantz MM, Ahmadiyeh N, Jia L, Herman P, Verzi MP, Doddapaneni H (2009). The 8q24 cancer risk variant rs6983267 shows long-range interaction with MYC in colorectal cancer. Nat Genet..

[64] The Cancer Genome Atlas (2012). Comprehensive genomic characterization of squamous cell lung cancers. Nature.

[65] Massion PP, Taflan PM, Jamshedur Rahman SM, Yildiz P, Shyr Y, Edgerton ME (2003). Significance of p63 amplification and overexpression in lung cancer development and prognosis. Cancer Res..

[66] Watanabe H, Ma Q, Peng S, Adelmant G, Swain D, Song W (2014). SOX2 and p63 colocalize at genetic loci in squamous cell carcinomas. J Clin Invest..

[67] Kandoth C, McLellan MD, Vandin F, Ye K, Niu B, Lu C (2013). Mutational landscape and significance across 12 major cancer types. Nature..

[68] Worsley Hunt R, Wasserman WW (2014). Non-targeted transcription factors motifs are a systemic component of ChIP-seq datasets. Genome Biol..

[69] Aguilera C, Nakagawa K, Sancho R, Chakraborty A, Hendrich B, Behrens A (2011). c-Jun N-terminal phosphorylation antagonises recruitment of the Mbd3/NuRD repressor complex. Nature.

[70] Tupler R, Perini G, Green MR (2001). Expressing the human genome. Nature..

[71] Razin SV, Borunova VV, Maksimenko OG, Kantidze OL (2012). Cys2His2 zinc finger protein family: classification, functions, and major members. Biochemistry (Mosc)..

[72] Vaquerizas JM, Kummerfeld SK, Teichmann SA, Luscombe NM (2009). A census of human transcription factors: function, expression and evolution. Nat Reviews Genetics..

[73] Reynisdottir I, Arason A, Einarsdottir BO, Gunnarsson H, Staaf J, Vallon-Christersson J (2013). High expression of ZNF703 independent of amplification indicates worse prognosis in patients with luminal B breast cancer. Cancer Med..

[74] Kang X, Chen W, Kim RH, Kang MK, Park NH (2009). Regulation of the hTERT promoter activity by MSH2, the hnRNPs K and D, and GRHL2 in human oral squamous cell carcinoma cells. Oncogene..

[75] Schaub MA, Boyle AP, Kundaje A, Batzoglou S, Snyder M (2012). Linking disease associations with regulatory information in the human genome. Genome Res..

[76] Maurano MT, Humbert R, Rynes E, Thurman RE, Haugen E, Wang H (2012). Systematic localization of common disease-associated variation in regulatory DNA. Science..

[77] Akhtar-Zaidi B, Cowper-Sal-lari R, Corradin O, Saiakhova A, Bartels CF, Balasubramanian D (2012). Epigenomic enhancer profiling defines a signature of colon cancer. Science..

[78] Hardison RC (2012). Genome-wide epigenetic data facilitate understanding of disease susceptibility association studies. J Biol Chem..

[79] Yao L, Tak YG, Berman BP, Farnham PJ (2014). Functional annotation of colon cancer risk SNPs. Nat Commun..

[80] Fredriksson NJ, Ny L, Nilsson JA, Larsson E (2014). Systematic analysis of noncoding somatic mutations and gene expression alterations across 14 tumor types. Nat Genet..

[81] Huang FW, Hodis E, Xu MJ, Kryukov GV, Chin L, Garraway LA (2013). Highly recurrent TERT promoter mutations in human melanoma. Science..

[82] Weinhold N, Jacobsen A, Schultz N, Sander C, Lee W (2014). Genome-wide analysis of noncoding regulatory mutations in cancer. Nat Genet..

[83] Hansen KD, Timp W, Bravo HC, Sabunciyan S, Langmead B, McDonald OG (2011). Increased methylation variation in epigenetic domains across cancer types. Nat Genet..

[84] BioConductor. [http://www.bioconductor.org/]

[85] ELMER source code. [https://github.com/lijingya/ELMER.git]

[86] ELMER usage vignette. [https://github.com/lijingya/ELMER/blob/master/vignettes/vignettes.pdf]

[87] ELMER user manual. [https://github.com/lijingya/ELMER/blob/master/inst/doc/ELMER_manual.pdf]

[88] TCGA data access. [https://tcga-data.nci.nih.gov/tcgafiles/ftp_auth/distro_ftpusers/anonymous/tumor/]

[89] TCGA pan-can analysis (Synapse).[https://www.synapse.org/#]

[90] Lawrence M, Huber W, Pages H, Aboyoun P, Carlson M, Gentleman R (2013). Software for computing and annotating genomic ranges. PLoS Comput Biol..

[91] Epigenomics Roadmap data access. [https://sites.google.com/site/epigenomeroadmapawg/project-updates/finalsignaltracksandalignmentfiles]

[92] FANTOM enhancer annotations. [http://enhancer.binf.ku.dk/presets/]

[93] Alexa A, Rahnenfuhrer J. topGO: Enrichment analysis for Gene Ontology. R package version 2180. 2010. [http://www.bioconductor.org/packages/release/bioc/html/topGO.html]

[94] Grant CE, Bailey TL, Noble WS (2011). FIMO: scanning for occurrences of a given motif. Bioinformatics..

[95] Wang J, Zhuang J, Iyer S, Lin X, Whitfield TW, Greven MC (2012). Sequence features and chromatin structure around the genomic regions bound by 119 human transcription factors. Genome Res..

[96] Shannon P. MotifDb: An annotated collection of Protein-DNA binding sequence motifs. Bioconductor. 2014, R package version 1.8.0. [http://www.bioconductor.org/packages/release/bioc/html/MotifDb.html]

[97] Ravasi T, Suzuki H, Cannistraci CV, Katayama S, Bajic VB, Tan K (2010). An atlas of combinatorial transcriptional regulation in mouse and man. Cell..

