# Supplementary figures and images for: Inferring regulatory element landscapes and transcription factor networks from cancer methylomes
Source: Genome Biol. 2015 May 21;16(1):105. doi: 10.1186/s13059-015-0668-3 (PMC4460959; doi:10.1186/s13059-015-0668-3)

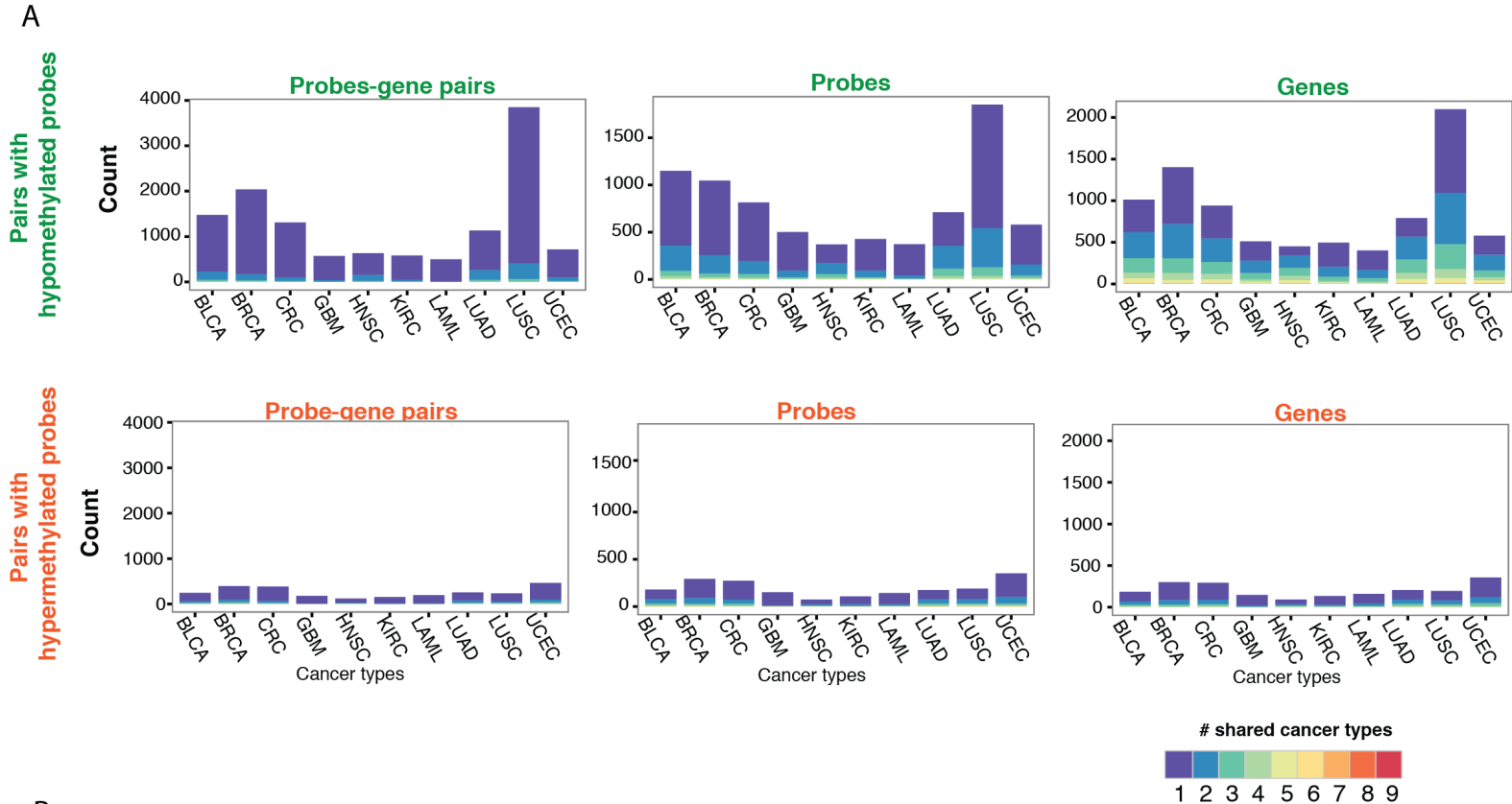

**B**

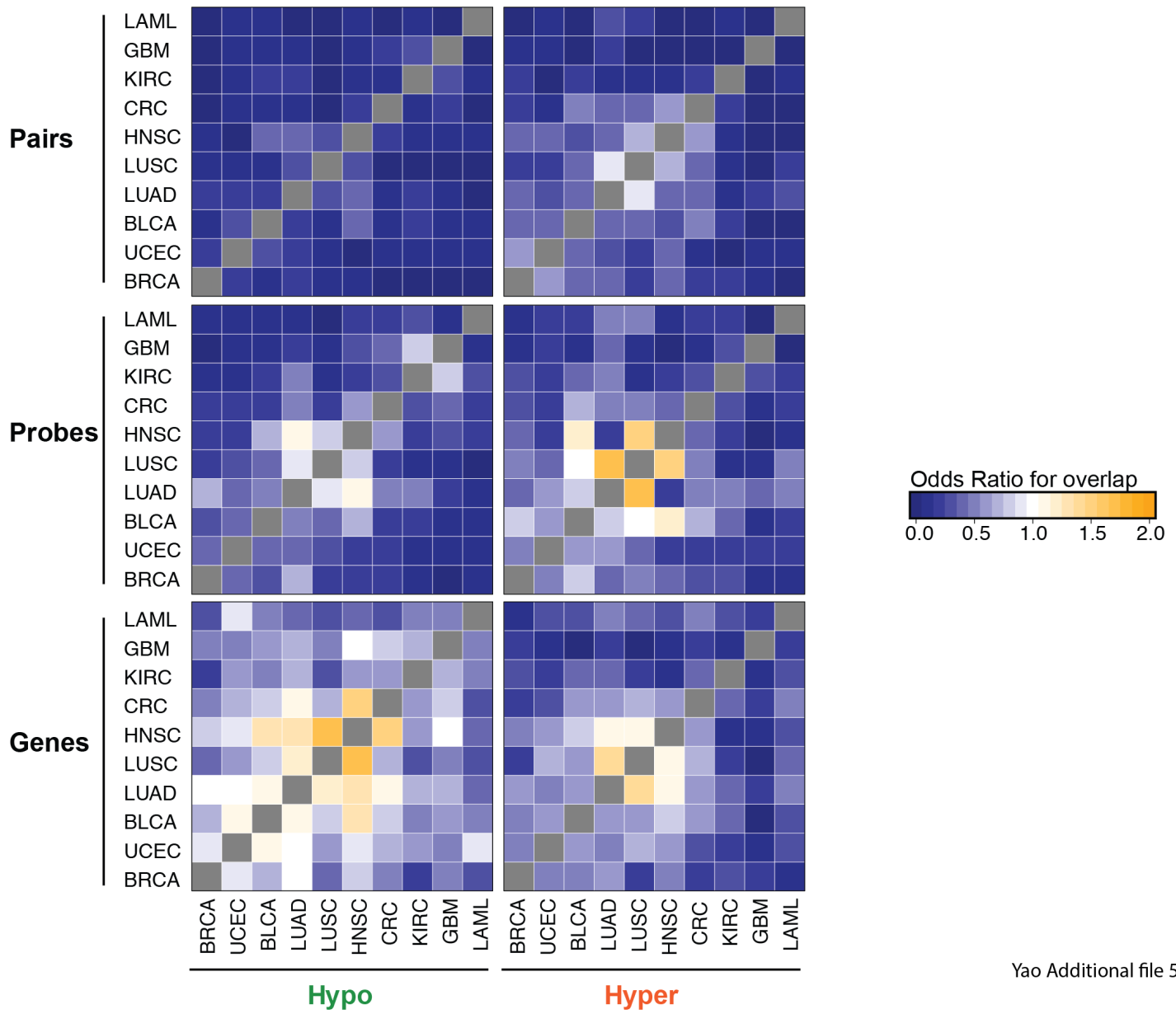

Supplement: Additional file 5: — Quantitative summary of links, probes, and genes for each cancer type. (A) Shown are histograms representing the number of putative probes-gene pairs, the number of total probes in the set of paired-probes, and the number of total genes in the set of paired probes for the set of hypomethylated (top) and hypermethylated (bottom) probe-gene pairs in each cancer type. For each plot, the number of probes identified in one or more tumor types is indicated by the colored bars. (B) Shown is a heatmap illustrating the similarity of probe-gene pairs, probes in the pairs, and genes in the pairs among the different cancer types. The color bar indicates the OR for the similarity (overlap) between the indicated cancer types (a higher OR indicates a more significant similarity). [file 13059_2015_668_MOESM5_ESM.pdf]

A

All  
significantly  
altered  
probes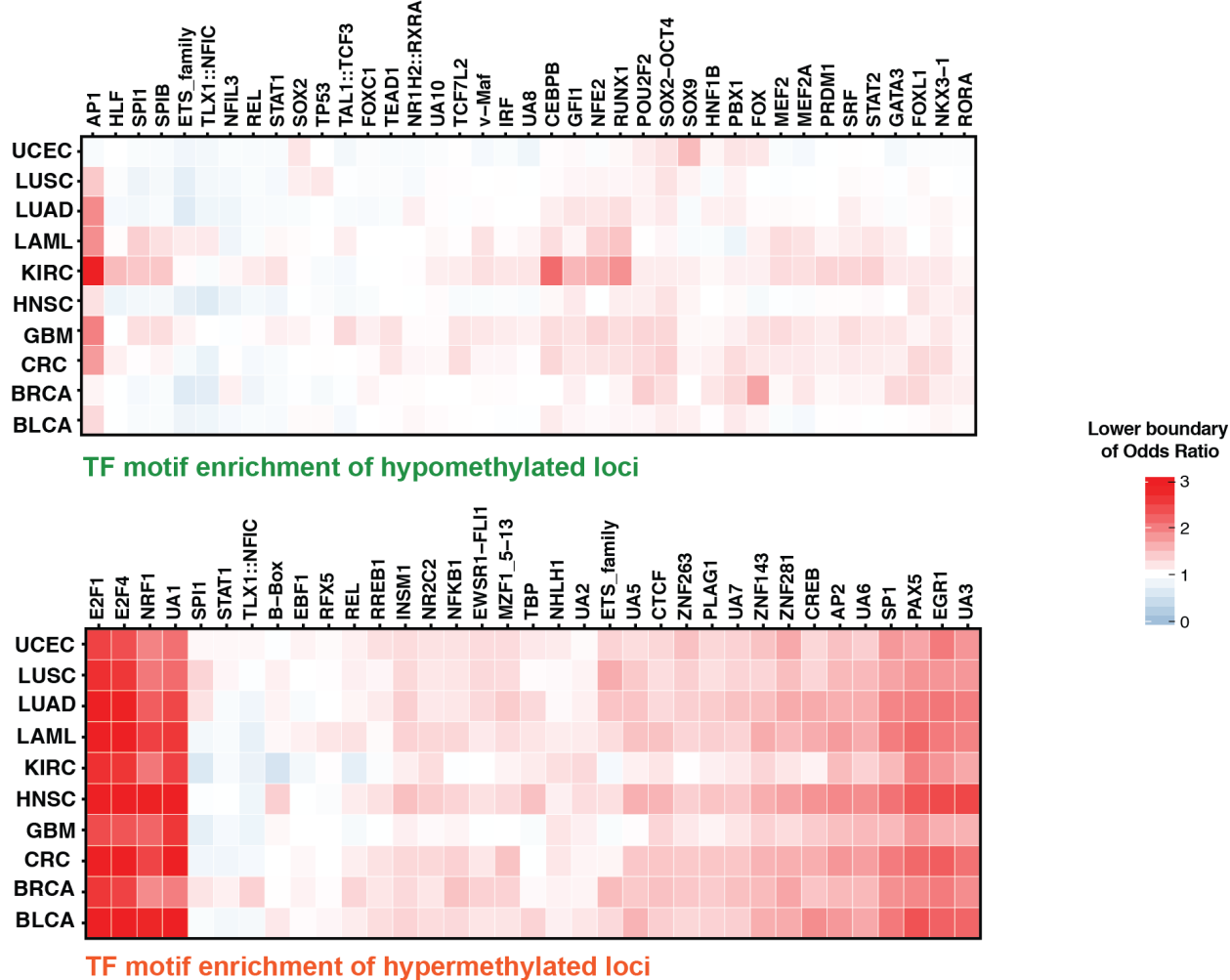

B

Probes in  
significant  
pairs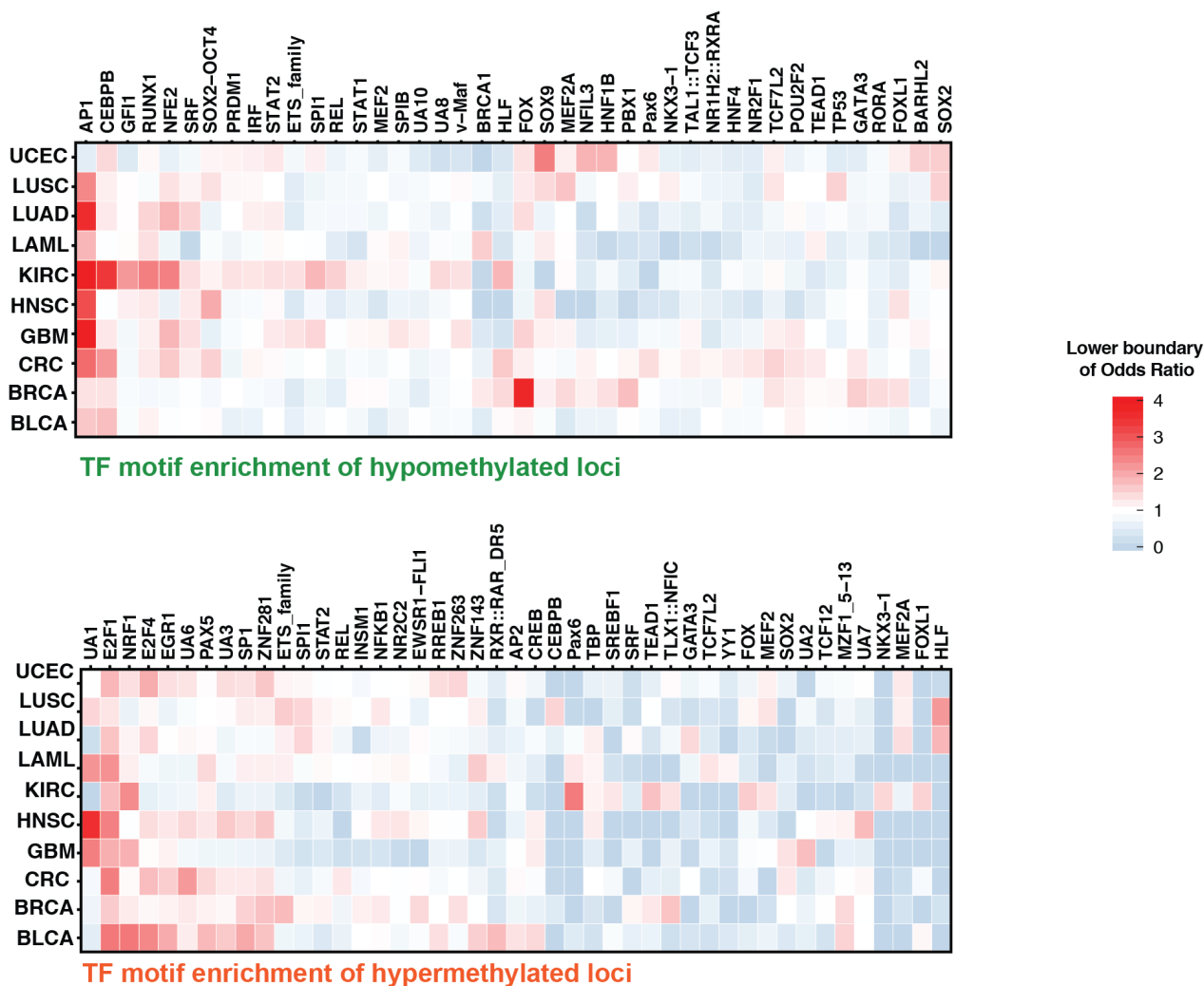

Supplement: Additional file 8: — Motif enrichment heatmaps. (A) Shown are the heatmaps for motifs that are enriched in the sets of all hypomethylated probes (top panel) and all hypermethylated probes (bottom panel). (B) Shown are the heatmaps for motifs that are enriched in the sets of only those hypomethylated (top panel) or hypermethylated (bottom panel) probes that are linked to putative target genes (B bottom panel). [file 13059_2015_668_MOESM8_ESM.pdf]

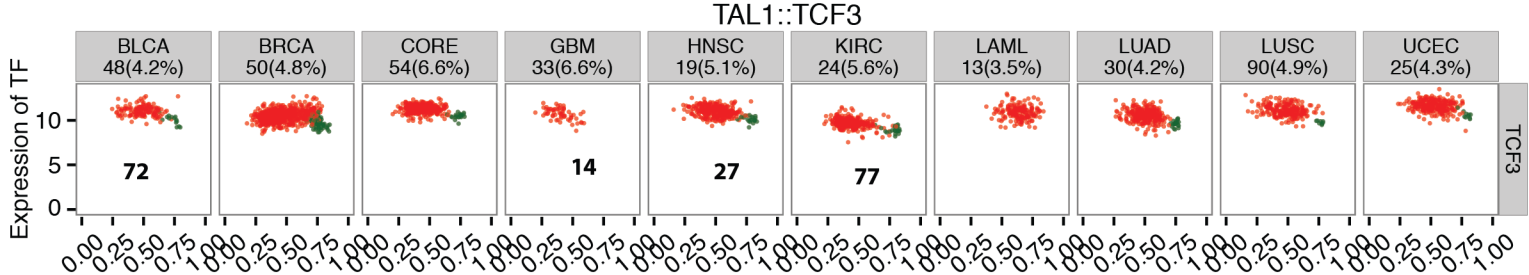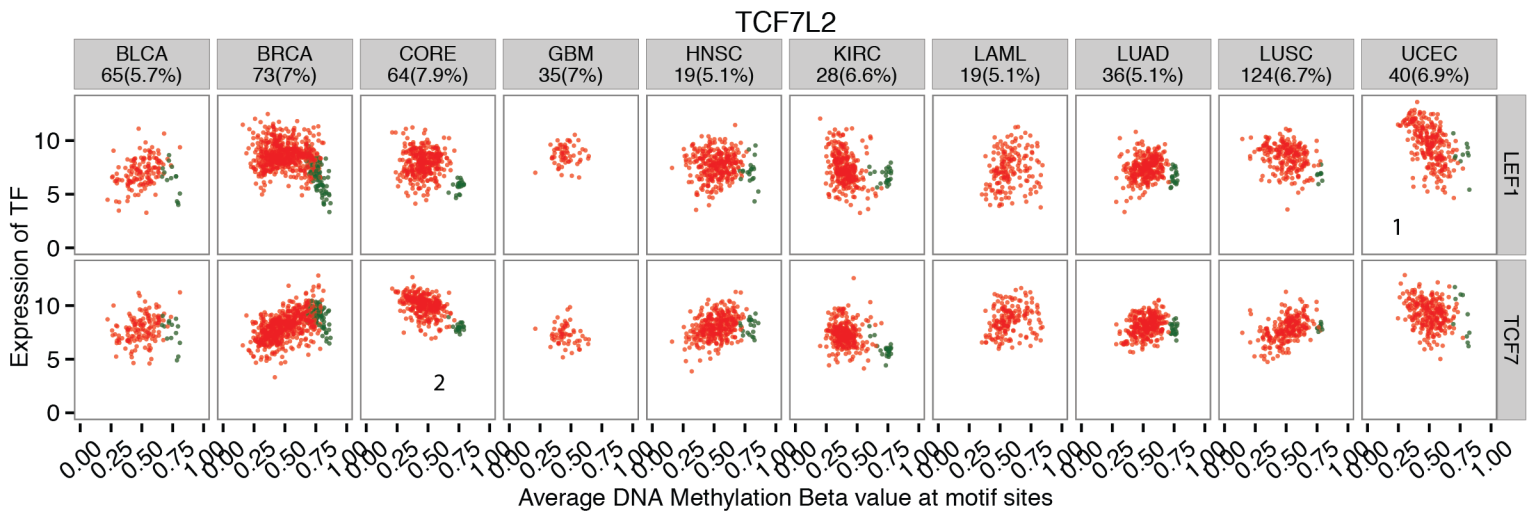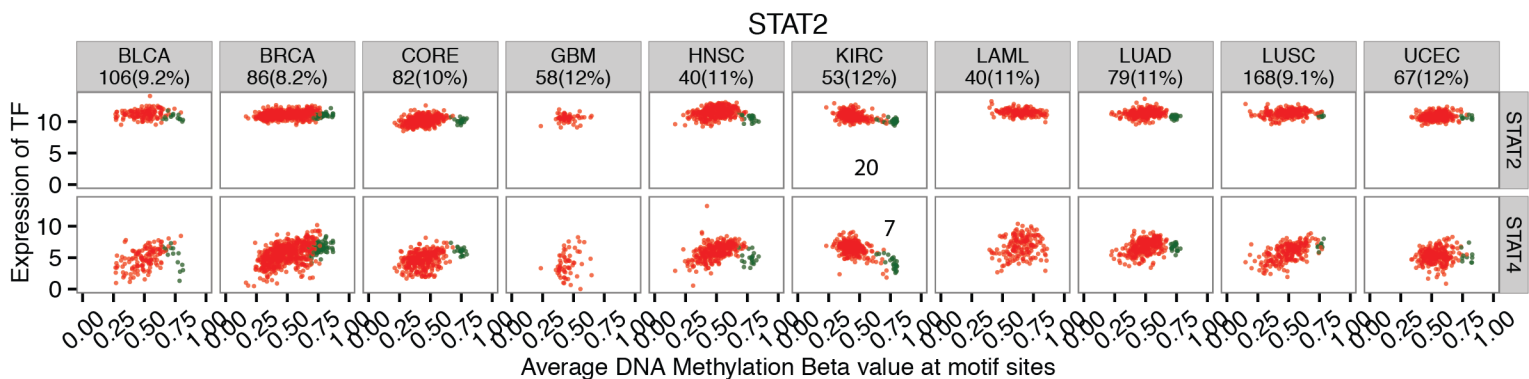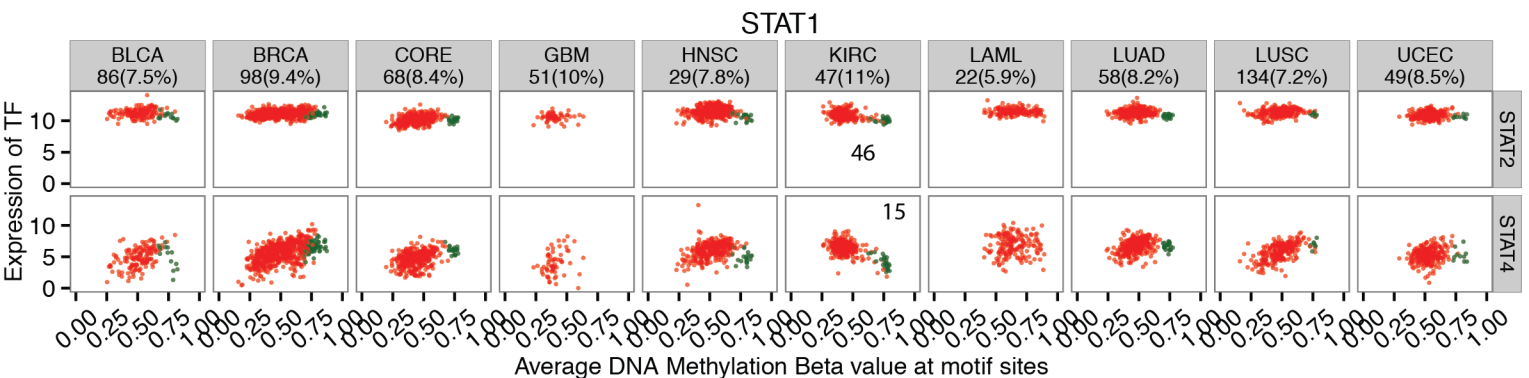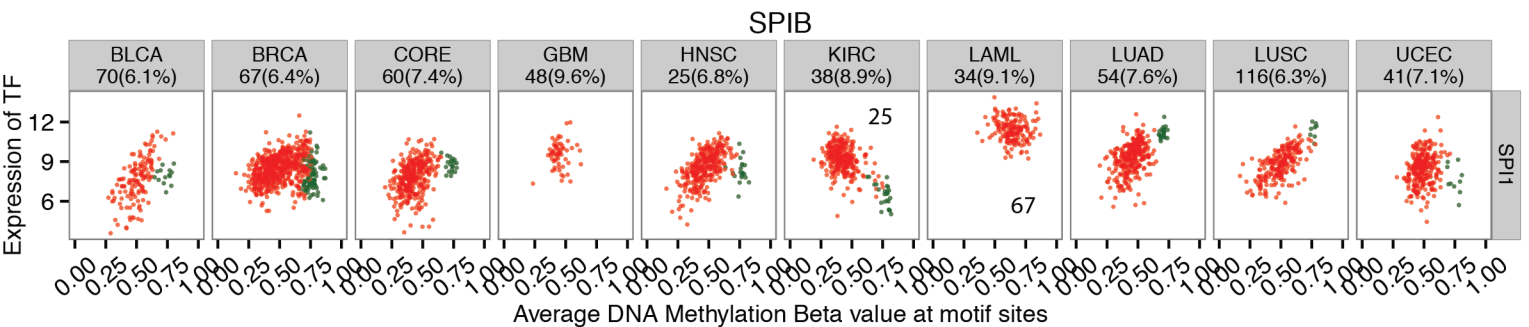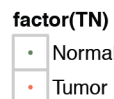

# SOX2-OCT4

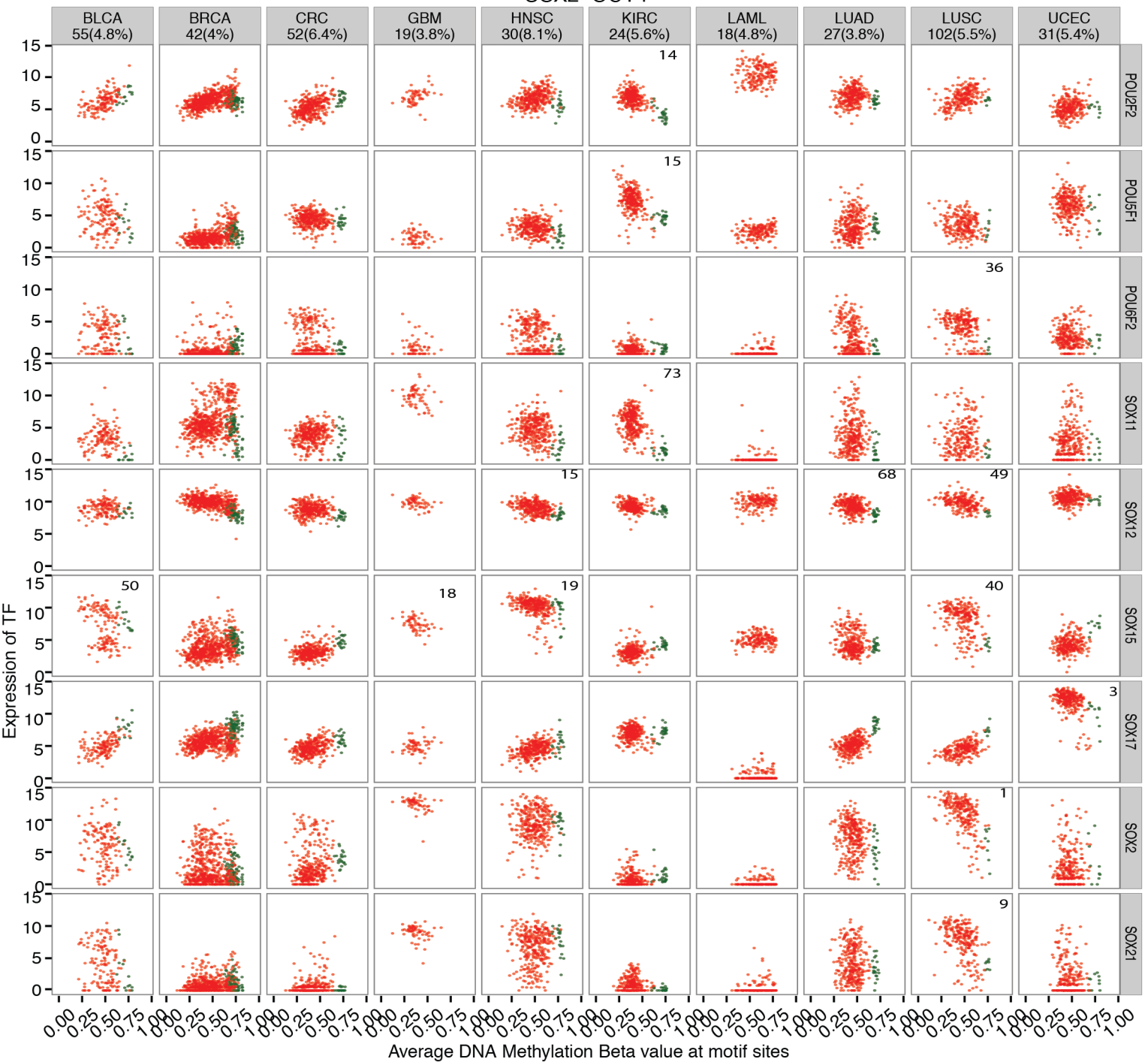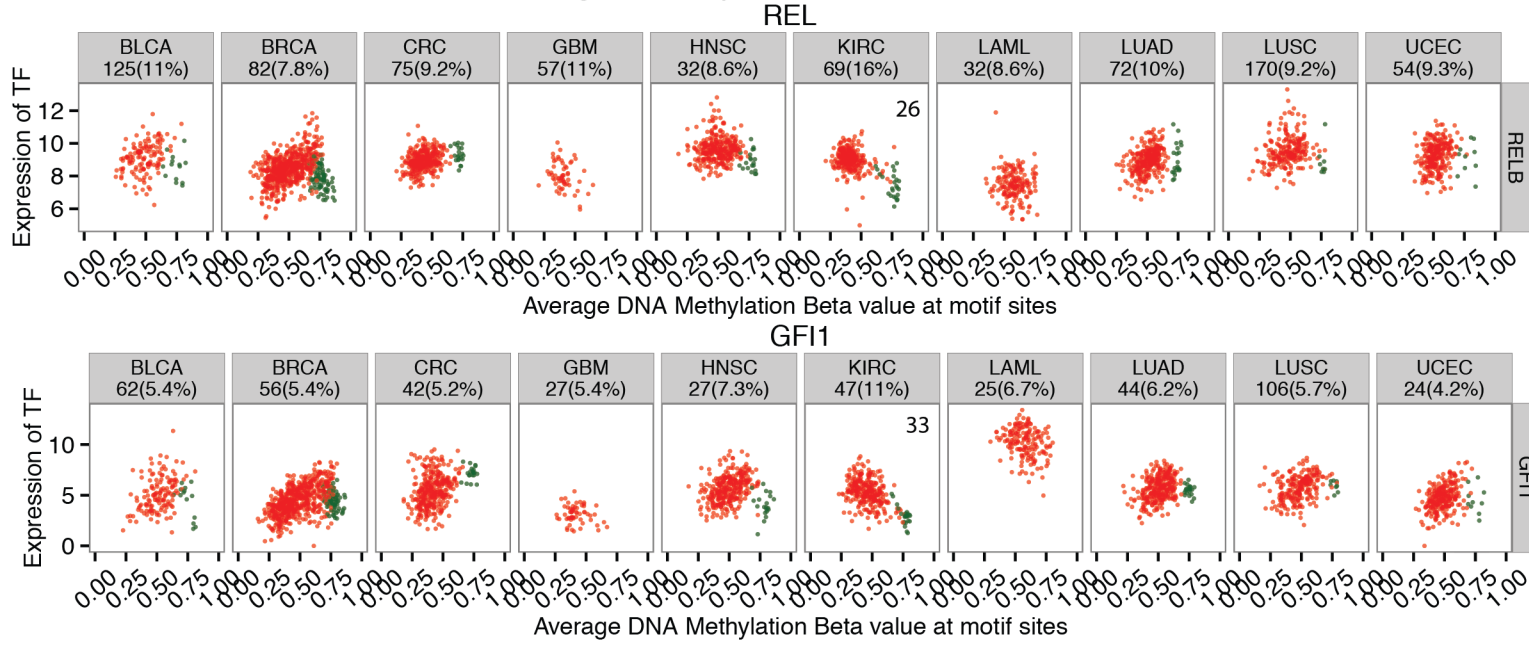

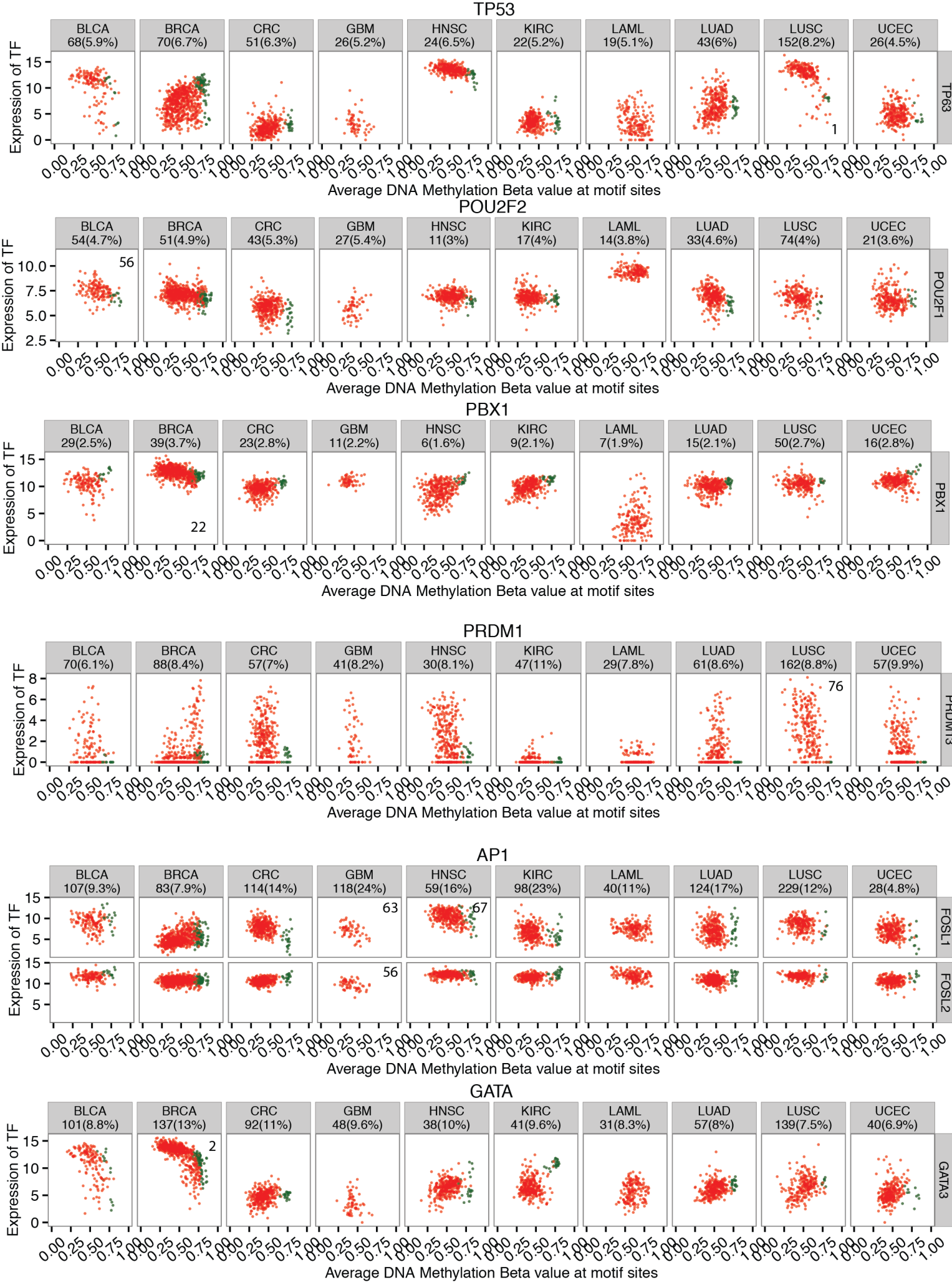

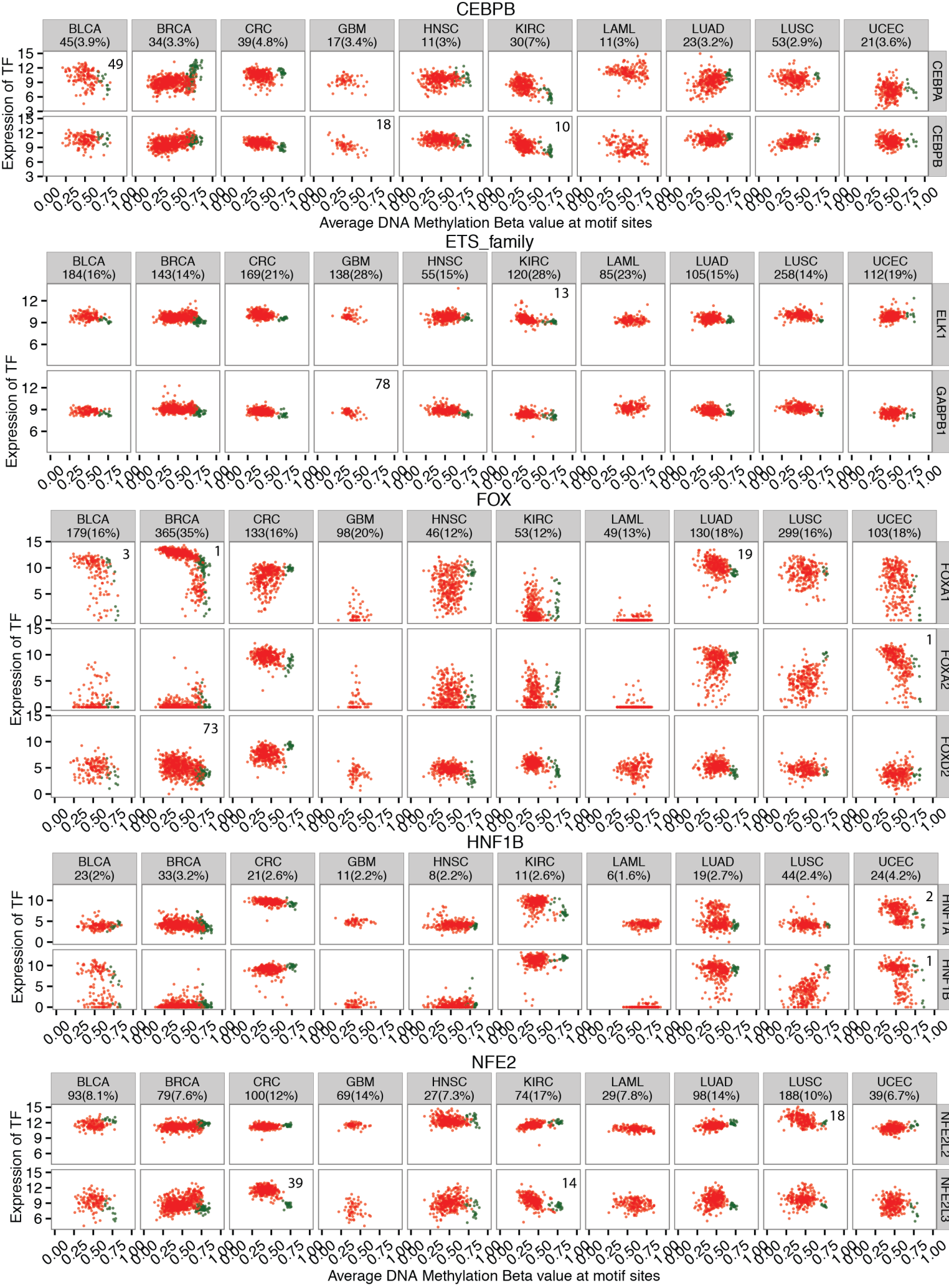

Supplement: Additional file 10: — Scatter plots for TF family members significantly associated with DNA methylation at distal enhancer regions having enriched motifs. Shown are scatter plots for average DNA methylation at probes having the indicated enriched motif (x axis, shown on the top of each set of panels) vs. the expression of the significantly correlated motif-relevant TF family members (y axis, shown on the right side of each panel). Each dot represents a different patient sample; red and green indicate the tumor and normal samples, respectively. Pairs that are within the top 5 % of TFs linked to a given motif are indicated with a number inside the cell. The number corresponds to the rank of the given TF relative to all 1,777 TFs (with ‘1’ being the most strongly correlated). [file 13059_2015_668_MOESM10_ESM.pdf]

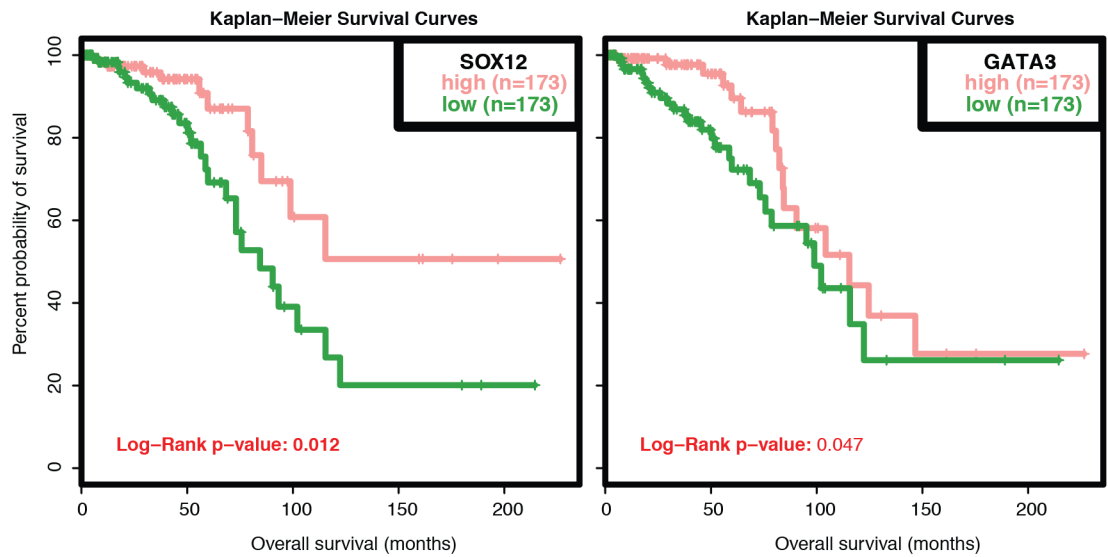

GBM

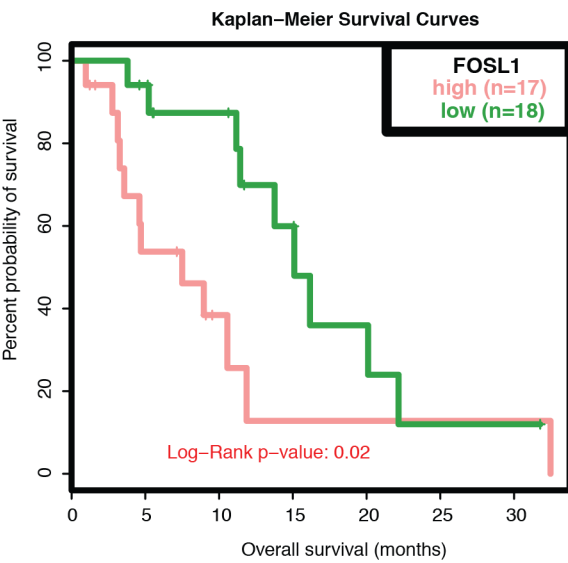

HNSC

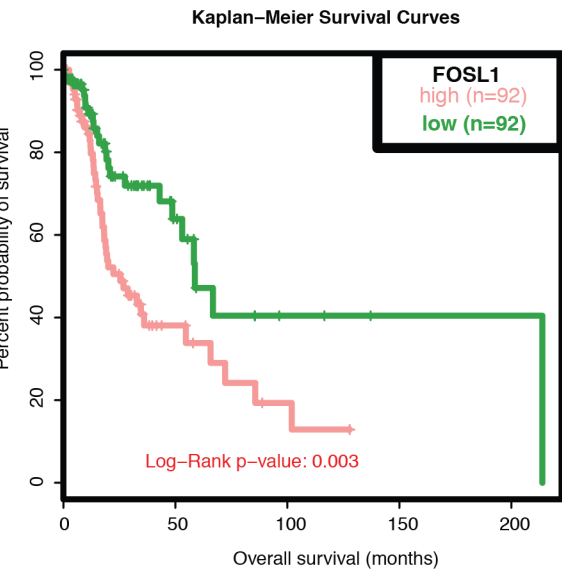

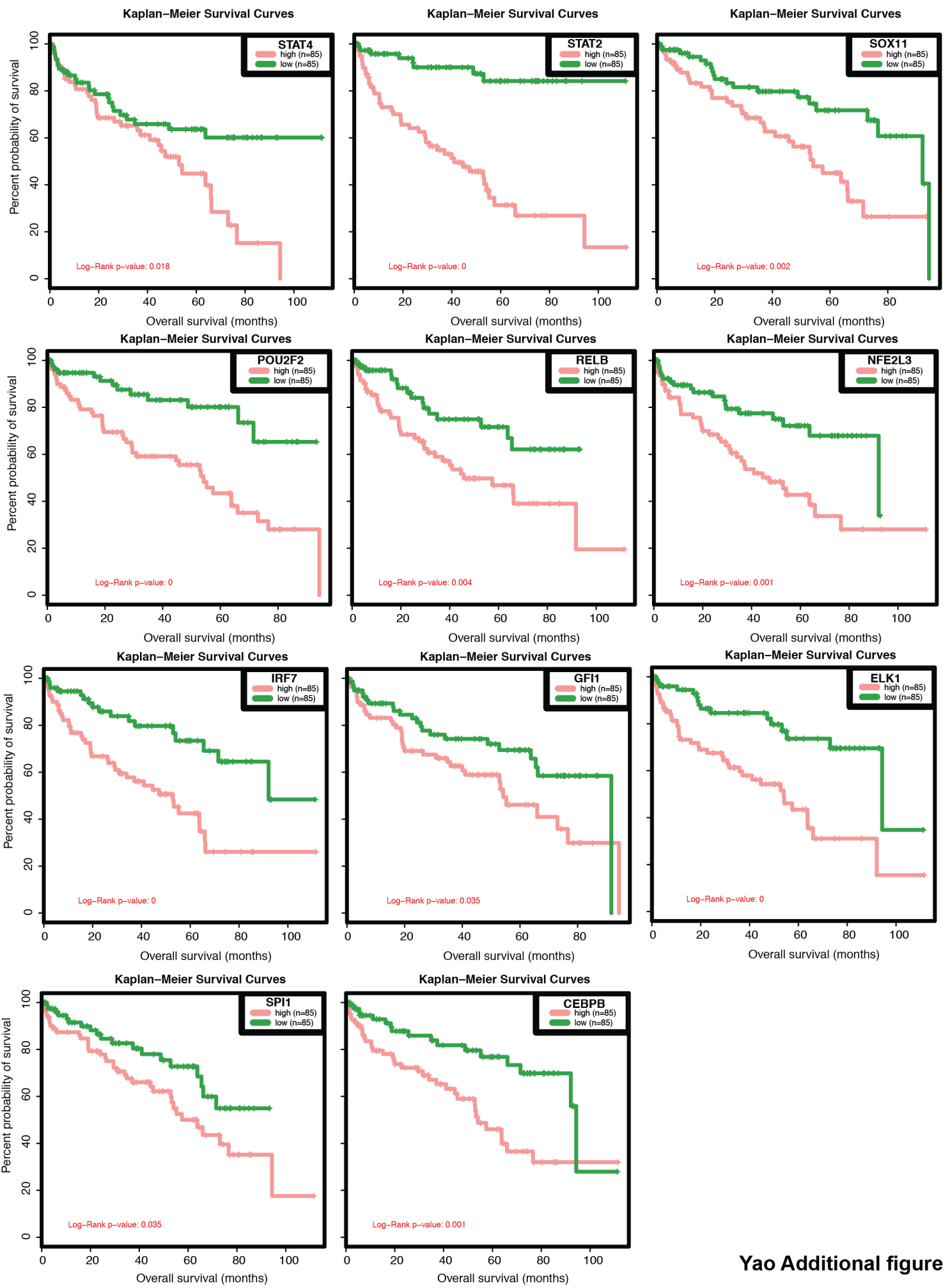

Supplement: Additional file 11: — Survival plots for TF family members significantly associated with DNA methylation at distal enhancer regions having enriched motifs. (A) The output of a Cox model regression analysis for the effects of expression of RUNX1 on survival within KIRC samples. Leukocyte methylation signature was calculated as in (PMID 22120008), and staging information was taken from TCGA clinical data. Leukocyte methylation signature was included to rule out RUNX1 expression from contaminating leukocytes, which are the main source of non-cancer cells in KIRC samples. (B) Kaplan-Meier survival curves for TF family members significantly associated with DNA methylation at the distal enhancer regions with enriched motifs in the indicated cancer type. The survival data for patients having tumors with the highest (top 30%) and lowest (bottom 30%) transcription factor expression are shown; the Log Rank test P value between the high and low groups is indicated. [file 13059_2015_668_MOESM11_ESM.pdf]

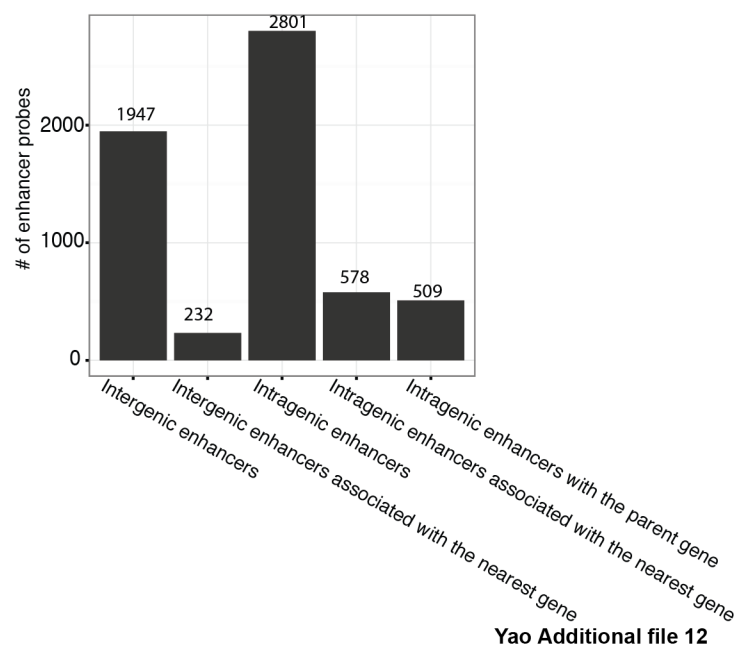

**Yao Additional file 12**

Supplement: Additional file 12: — Proportion of intragenic vs. intergenic enhancers that regulate the nearest gene. Shown are bar graphs indicating the number of intergenic vs. intragenic enhancers, the number of each category that is associated with expression of the nearest gene, and the number of intragenic enhancers associated with expression of the nearest gene with that gene being the one in which the enhancer resides. [file 13059_2015_668_MOESM12_ESM.pdf]

Yao Additional file 14

A

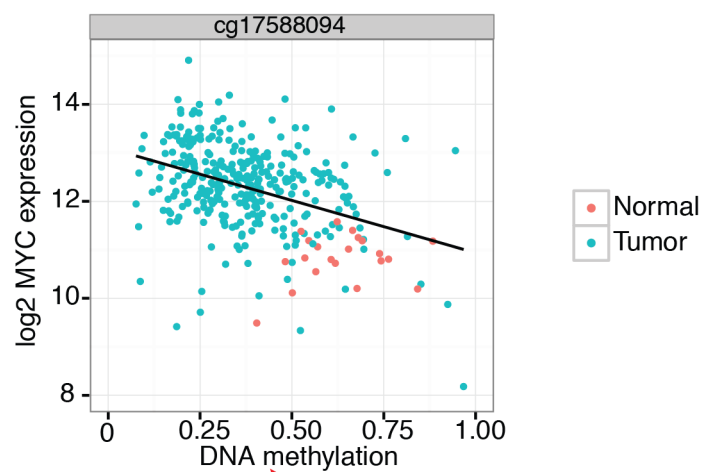

B

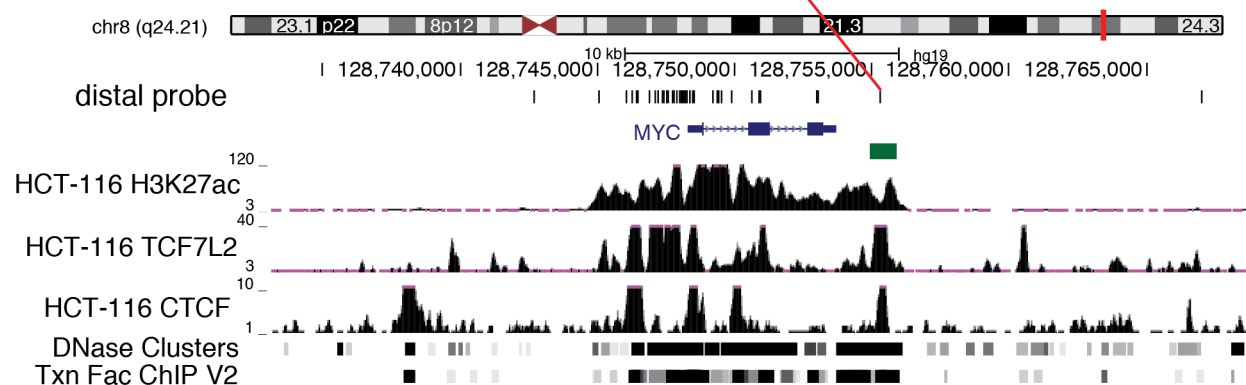

Supplement: Additional file 14: — MYC 3’ end enhancer regulates MYC expression in colorectal cancer tissue. (A) Shown is a scatter plot showing DNA methylation at probes located at the 3’ end of the MYC gene vs. the expression of MYC RNA. Each dot represents a different patient sample; red and green indicate the tumor and normal samples, respectively. (B) Shown is the location of the MYC 3’ enhancer and the ENCODE ChIP-seq histone and transcription factor tracks from the University of California, Santa Cruz genome browser. The green bar indicates the location of enhancer that has been previously identified to regulate MYC expression in the HCT116 colon cancer cell line [57, 58]. [file 13059_2015_668_MOESM14_ESM.pdf]
