# Supplementary material for: Inferring regulatory element landscapes and transcription factor networks from cancer methylomes
Source: Genome Biol. 2015 May 21;16(1):105. doi: 10.1186/s13059-015-0668-3 (PMC4460959; doi:10.1186/s13059-015-0668-3)
Supplement: Additional file 6: — Rank of putative target gene according to distance in the enhancer-gene pairs for each cancer type. (A) Shown is the distribution for the ranking (by distance) of each putative target gene linked to an enhancer for enhancers that are significantly associated with more than one gene. (B) Shown is the distribution for the ranking (by distance) of each putative target gene linked to an enhancer for each cancer type. The left panel shows the pairs for which the enhancer is significantly associated with more than one gene and the right panel shows the pairs for which the enhancer is significantly associated with only one gene. [file 13059_2015_668_MOESM6_ESM.pdf]

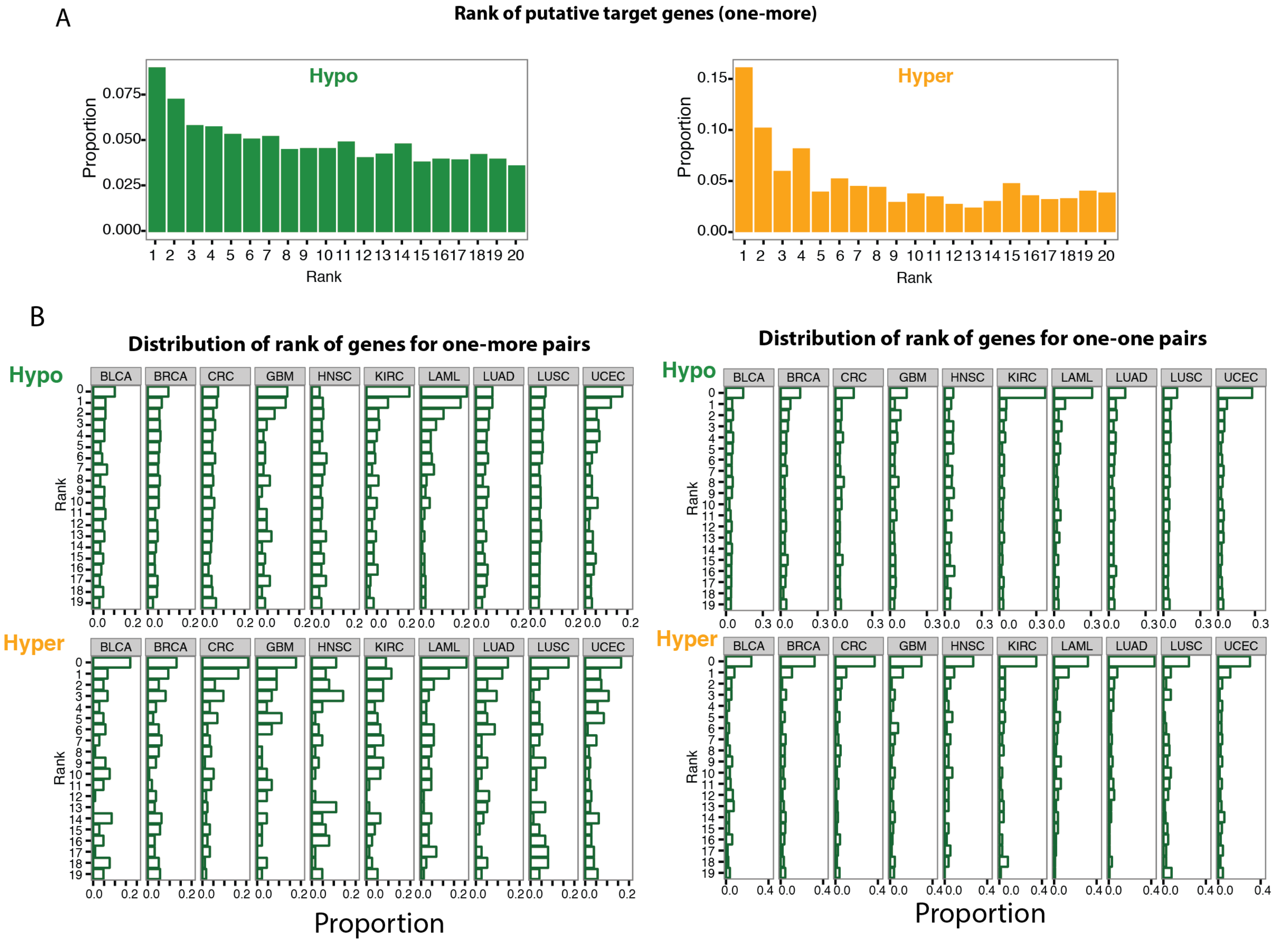

Yao Additional file 6
