# Supplementary material for: Inferring regulatory element landscapes and transcription factor networks from cancer methylomes
Source: Genome Biol. 2015 May 21;16(1):105. doi: 10.1186/s13059-015-0668-3 (PMC4460959; doi:10.1186/s13059-015-0668-3)
Supplement: Additional file 9: — Plots of association between all human TFs and DNA methylation at enriched motif sites. Shown are TF ranking plots based on the score (-log10(Pr)) of association between TF expression and DNA methylation of the motif in the cancer type in which the motifs are enriched. The dashed blue line indicates the boundary of the top 5 % association score. The top three associated TFs and the TF family members (dots in red) that are associated with that specific motif are labeled in the plot. [file 13059_2015_668_MOESM9_ESM.pdf]

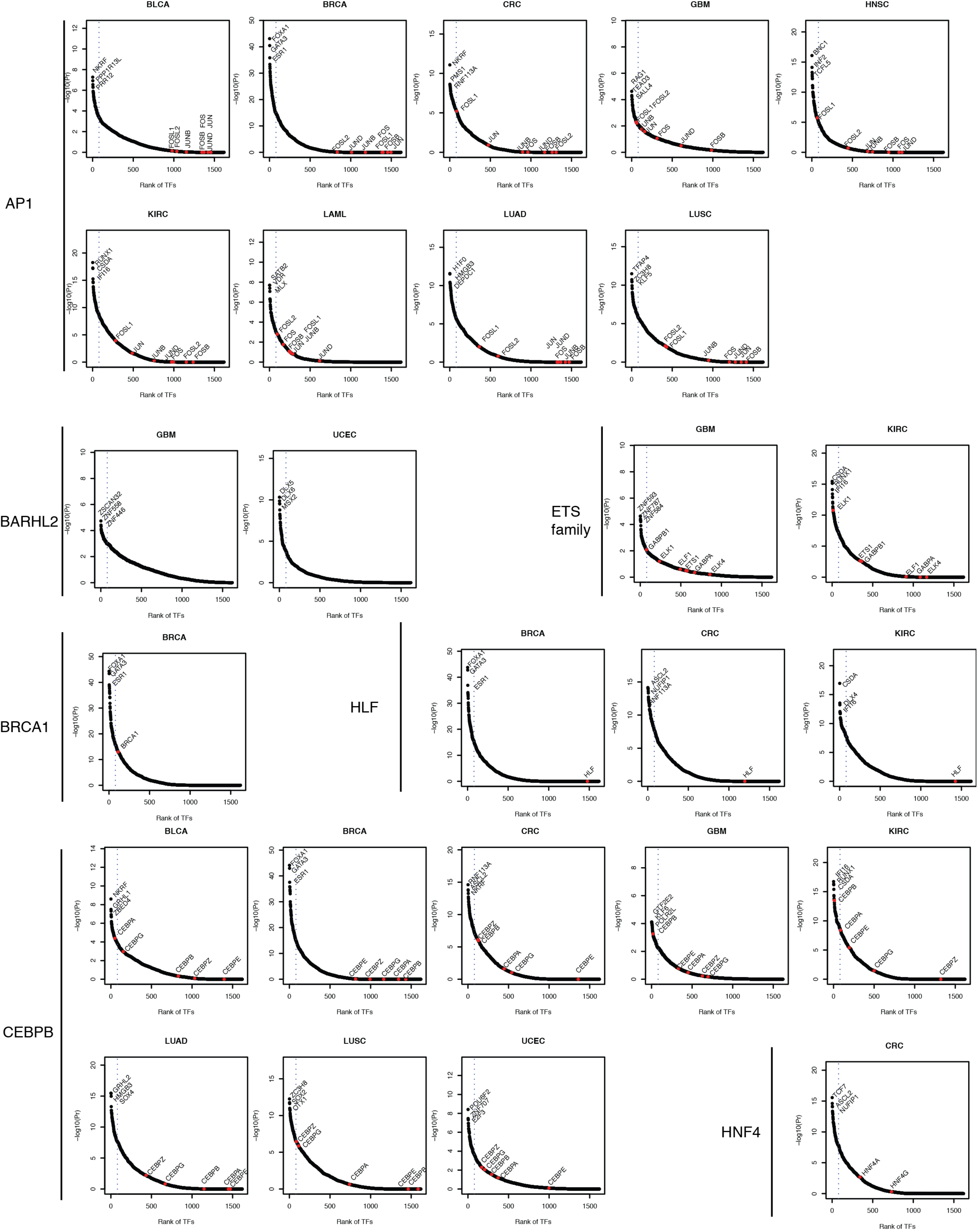

AP1
BLCA
NKRF
PPP1R13L
PRR12
FOSL1
FOSL2
JUNB
FOSB
FOS
JUND
JUN
BRCA
FOXA1
GATA3
ESR1
CRC
PMS1
RNF113A
GBM
RAG1
TEAD3
SALL4
HNSC
BNC1
KLF5
KIRC
RUNX1
CSDA
IFI16
LAML
SATB2
VDR
MLX
LUAD
H1F0
HMGB3
DEPDC1
LUSC
TFAP4
ZC3H8
BARHL2
ZSCAN32
ZNF558
ZNF446
UCEC
DLX5
DLX6
MSX2
ETS family
ZNF593
ZNF787
ZNF584
GABPB1
ELK1
ELF1
ETS1
GABPA
ELK4
BRCA1
HLF
ASCL2
NUFIP1
DLX4
CEBPB
GRHL1
ZBED4
CEBPA
CEBPG
CEBPZ
CEBPE
GTF2E2
POLR2L
GRHL2
SOX4
OTX1
POU6F2
E2F3
HNF4
TCF7
HNF4A
HNF4G
−log10(Pr)
Rank of TFs

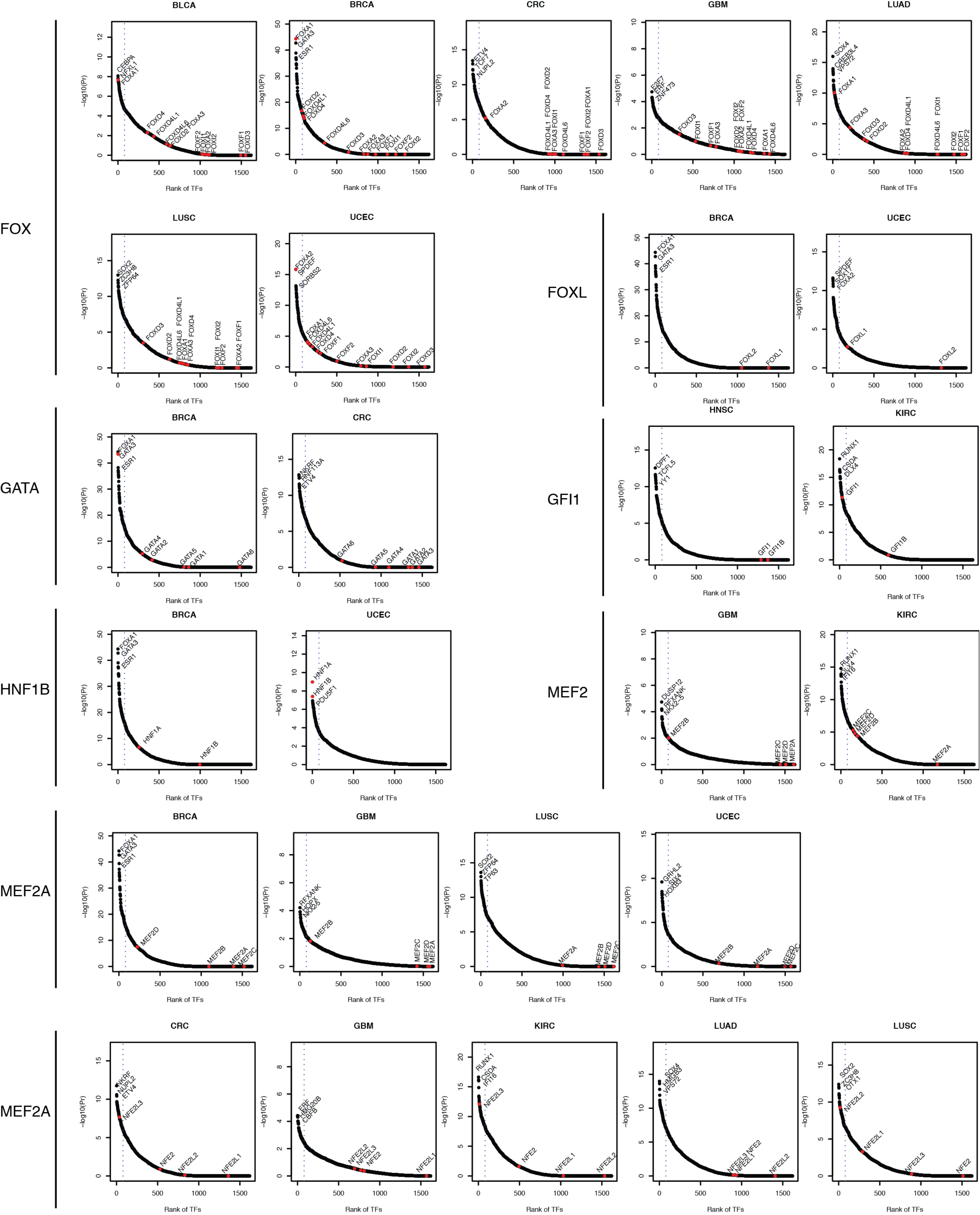

FOX
BLCA
CEBPA
NFXL1
FOXA1
FOXD4
FOXD4L1
FOXD4L6
FOXD2
FOXA3
FOXF2
FOXI1
FOXA2
FOXI2
FOXF1
FOXD3
BRCA
FOXA1
GATA3
ESR1
FOXD2
FOXD4L1
FOXD4
FOXD4L6
CRC
ETV4
TCF7
NUPL2
FOXA2
GBM
E2F7
ERF
ZNF473
LUAD
SOX4
CREB3L4
VPS72
LUSC
SOX2
ZC3H8
ZFP64
UCEC
FOXA2
SPDEF
SORBS2
FOXL
FOXL2
FOXL1
GATA
NKRF
PNF113A
GATA4
GATA2
GATA5
GATA1
GATA6
GFI1
HNSC
DPF1
TCFL5
YY1
GFI1B
KIRC
RUNX1
CSDA
DLX4
HNF1B
HNF1A
POU5F1
MEF2
DUSP12
RFXANK
NKX2-5
MEF2B
MEF2C
MEF2D
MEF2A
IFI16
MEF2A
GRHL2
SIX4
HOXB3
TP63
NFE2L3
NFE2
NFE2L2
NFE2L1
HMG20B
CBFB
HMGB3
OTX1
−log10(Pr)
Rank of TFs

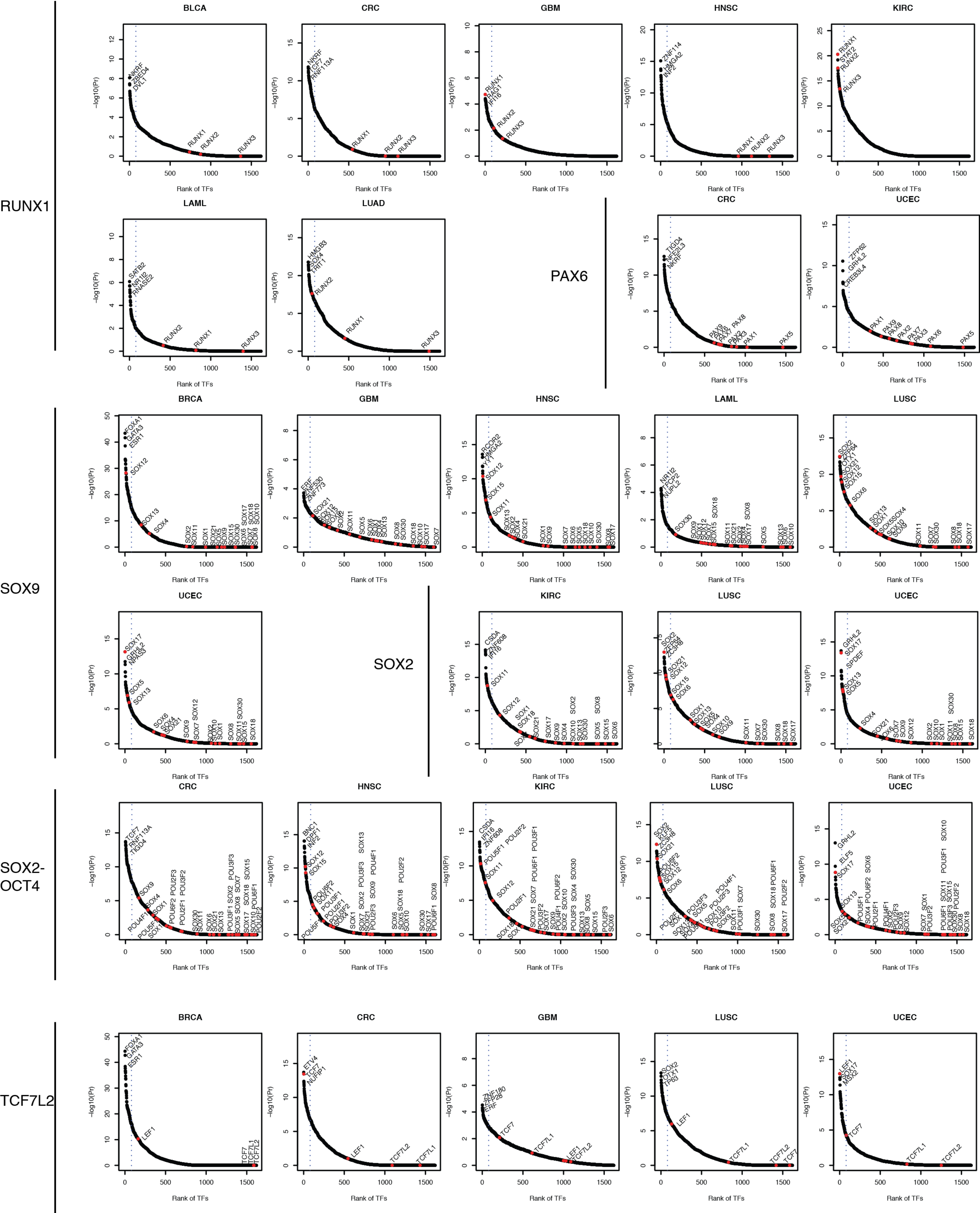
RUNX1
BLCA
NKRF
ZBED4
DVL1
RUNX1
RUNX2
RUNX3
CRC
NKRF
TCF7
RNF113A
RUNX1
RUNX2
RUNX3
GBM
RUNX1
GAGT
IFI16
RUNX2
RUNX3
HNSC
ZNF114
HMGA2
INF2
RUNX1
RUNX2
RUNX3
KIRC
RUNX1
STAT2
RUNX2
RUNX3
LAML
SATB2
NR1I2
RNASE2
RUNX2
RUNX1
RUNX3
LUAD
HMGB3
SOX4
TRIT1
RUNX2
RUNX1
RUNX3
PAX6
CRC
TIGD4
NFE2L3
NKRF
PAX9
PAX6
PAX7
PAX8
PAX2
PAX3
PAX1
PAX5
UCEC
ZFP62
GRHL2
CREB3L4
PAX1
PAX9
PAX8
PAX2
PAX7
PAX3
PAX6
PAX5
SOX9
BRCA
FOXA1
GATA3
ESR1
SOX12
SOX13
SOX4
GBM
ERF
ZNF630
ZNF773
HNSC
RCOR2
HMGA2
YY1
SOX12
SOX15
SOX11
LAML
NR1I2
TFCP2
NUPL2
SOX30
SOX18
SOX8
LUSC
SOX2
SOX6
UCEC
SOX17
GRHL2
NPAS3
SOX5
SOX13
SOX2
KIRC
CSDA
ZNF608
IFI16
SOX11
SOX12
LUSC
UCEC
GRHL2
SOX17
SPDEF
SOX13
SOX5
SOX2-OCT4
CRC
TCF7
RNF113A
TIGD4
HNSC
BNC1
INF2
KIRC
CSDA
IFI16
ZNF608
POU5F1
POU2F2
POU3F1
LUSC
UCEC
GRHL2
ELF5
SOX17
TCF7L2
BRCA
FOXA1
GATA3
ESR1
LEF1
TCF7
TCF7L1
TCF7L2
CRC
ETV4
TCF7
NUFIP1
LEF1
TCF7L2
TCF7L1
GBM
ZNF180
ZFP28
ERF
TCF7
TCF7L1
LEF1
TCF7L2
LUSC
SOX2
OTX1
TP63
LEF1
TCF7L1
TCF7L2
TCF7
UCEC
LEF1
SOX17
MSX2
TCF7
TCF7L1
TCF7L2
−log10(Pr)
Rank of TFs

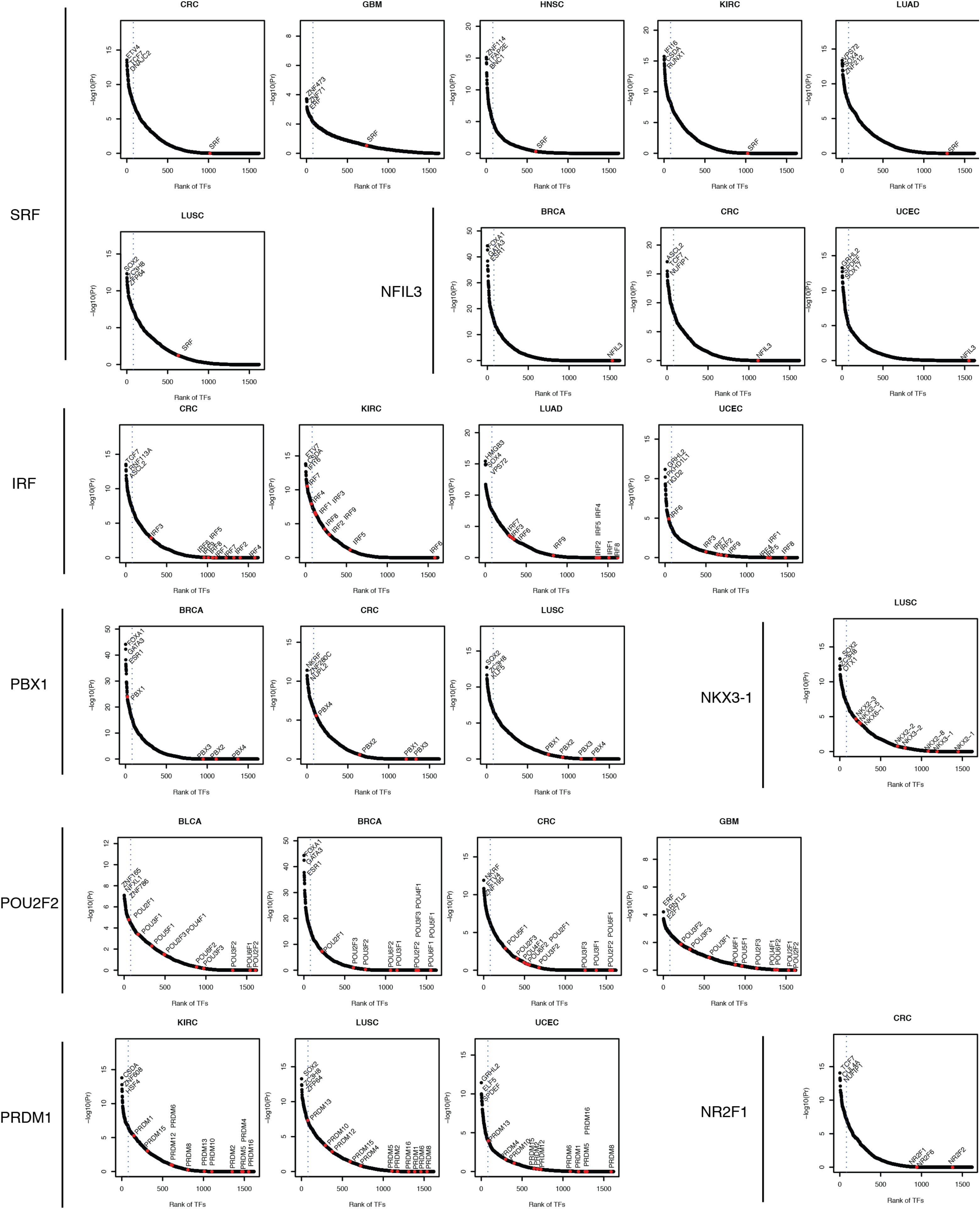
SRF
CRC
ETV4
TCF7
DNAJC2
SRF
GBM
ZNF473
ZNF71
ERF
SRF
HNSC
ZNF114
TFAP2E
BNC1
SRF
KIRC
IFI16
CSDA
RUNX1
SRF
LUAD
VPS72
SOX4
ZNF212
SRF
LUSC
SOX2
ZC3H8
ZFP64
SRF
NFIL3
BRCA
FOXA1
GATA3
ESR1
NFIL3
CRC
ASCL2
TCF7
NUFIP1
NFIL3
UCEC
GRHL2
SPDEF
SOX17
NFIL3
IRF
CRC
TCF7
RNF113A
ASCL2
IRF3
IRF6
IRF8
IRF5
IRF1
IRF7
IRF2
IRF4
KIRC
ETV7
CSDA
IFI16
IRF7
IRF4
IRF1
IRF3
IRF8
IRF9
IRF2
IRF5
IRF6
LUAD
HMGB3
SOX4
VPS72
IRF7
IRF3
IRF6
IRF9
IRF4
IRF5
IRF2
IRF1
IRF8
UCEC
GRHL2
PKHD1L1
TIGD2
IRF6
IRF3
IRF7
IRF2
IRF9
IRF1
IRF4
IRF5
IRF8
PBX1
BRCA
FOXA1
GATA3
ESR1
PBX1
PBX3
PBX2
PBX4
CRC
NKRF
ZNF280C
NUPL2
PBX4
PBX2
PBX1
PBX3
LUSC
SOX2
ZC3H8
KLF5
PBX1
PBX2
PBX3
PBX4
NKX3-1
LUSC
SOX2
ZC3H8
OTX1
NKX2−3
NKX2−5
NKX6−1
NKX2−2
NKX3−2
NKX2−8
NKX3−1
NKX2−1
POU2F2
BLCA
ZNF165
NFXL1
ZNF786
POU2F1
POU3F1
POU5F1
POU2F3
POU4F1
POU6F2
POU3F3
POU3F2
POU6F1
POU2F2
BRCA
FOXA1
GATA3
ESR1
POU2F1
POU2F3
POU3F2
POU6F2
POU3F1
POU3F3
POU4F1
POU2F2
POU6F1
POU5F1
CRC
NKRF
ETV4
ZNF195
POU5F1
POU2F3
POU4F1
POU6F2
POU3F2
POU2F1
POU3F3
POU3F1
POU2F2
POU6F1
GBM
ERF
ARNTL2
E2F7
POU3F2
POU3F3
POU3F1
POU6F1
POU5F1
POU2F3
POU4F1
POU6F2
POU2F1
POU2F2
PRDM1
KIRC
CSDA
ZNF608
HSF4
PRDM1
PRDM15
PRDM12
PRDM6
PRDM8
PRDM13
PRDM10
PRDM2
PRDM5
PRDM4
PRDM16
LUSC
SOX2
ZC3H8
ZFP64
PRDM13
PRDM10
PRDM12
PRDM15
PRDM4
PRDM5
PRDM2
PRDM16
PRDM1
PRDM6
PRDM8
UCEC
GRHL2
ELF5
SPDEF
PRDM13
PRDM4
PRDM10
PRDM15
PRDM2
PRDM12
PRDM6
PRDM1
PRDM5
PRDM16
PRDM8
NR2F1
CRC
TCF7
CUL4A
NUFIP1
NR2F1
NR2F6
NR2F2
−log10(Pr)
Rank of TFs

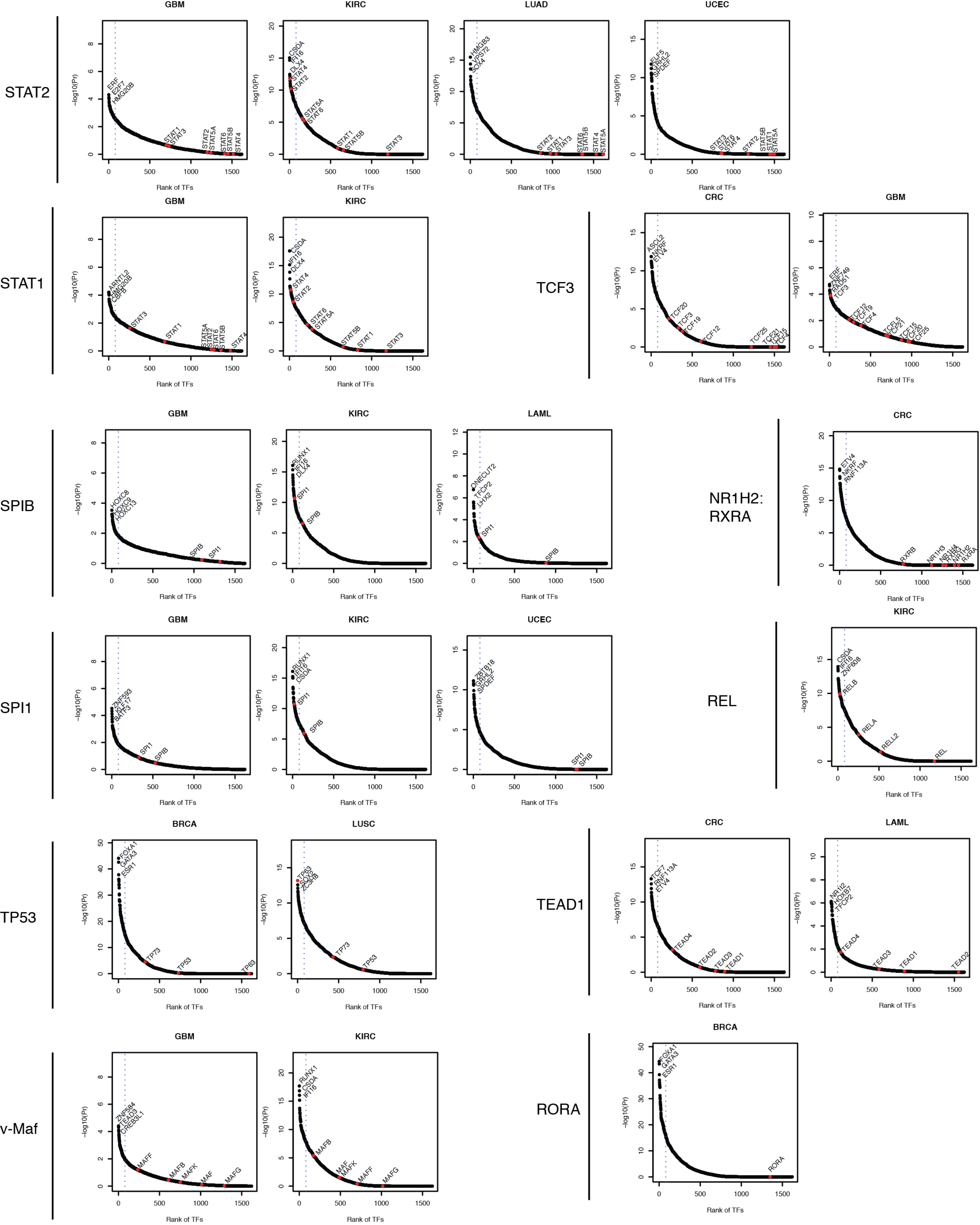

STAT2
GBM
KIRC
LUAD
UCEC
STAT1
GBM
KIRC
TCF3
CRC
GBM
SPIB
GBM
KIRC
LAML
NR1H2:
RXRA
CRC
SPI1
GBM
KIRC
UCEC
REL
KIRC
TP53
BRCA
LUSC
TEAD1
CRC
LAML
v-Maf
GBM
KIRC
RORA
BRCA
-log10(Pr)
Rank of TFs
