# Supplementary material for: Inferring regulatory element landscapes and transcription factor networks from cancer methylomes
Source: Genome Biol. 2015 May 21;16(1):105. doi: 10.1186/s13059-015-0668-3 (PMC4460959; doi:10.1186/s13059-015-0668-3)

Yao_Additional File 16

TF	Cancer Type	Survival	logRankP
ZNF114	HNSC (6)	high=worse survival	0.018
ZNF365	KIRC (9)	high=worse survival	0
ZNF683	KIRC (9)	high=worse survival	0.001
ZNF92	CRC (4)	high=worse survival	0.012
ZNF273	CRC (4)	high=worse survival	0.035
ZNF804A	LAML (3)	high=better survival	0.023
ZNF773	GBM (3)	high=better survival	0.02
ZNF593	BLCA (4)	high=better survival	0.007
ZNF165	BLCA (4)	high=better survival	0.019
ZNF77	LUSC (11)	high=better survival	0.019
ZNF414	GBM (4)	high=better survival	0.027

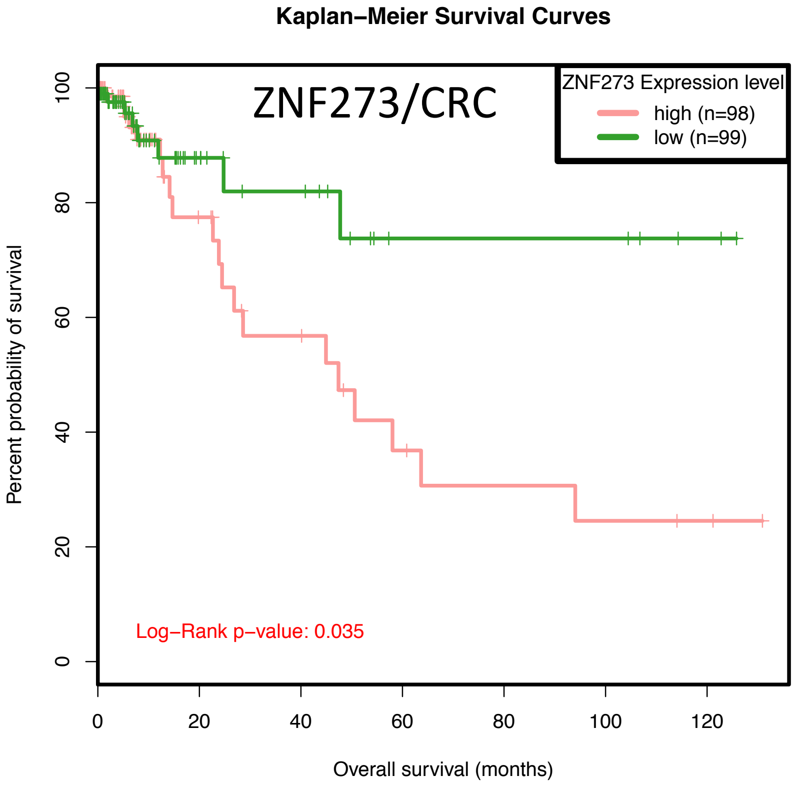

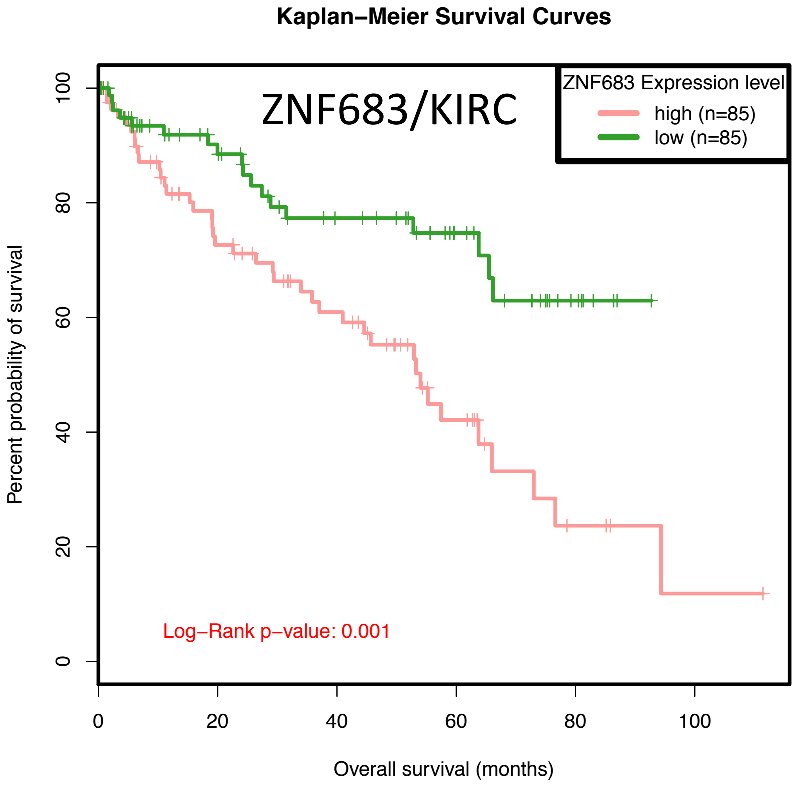

Supplement: Additional file 16: — Survival analysis of commonly identified ZNFs. (A) Shown is a table listing the subset of ZNFs (the entire list can be found in Additional file 17) which were identified in the top 1 % of ranked TFs, which were significantly associated with multiple different motifs in a specific cancer type (the number of motifs with which the TF was associated is listed in parentheses), and whose expression level significantly correlates with patient survival. The direction of correlation is labeled in column labeled ‘Survival’ (red and green color represents high expression correlated with worse survival or better bad survival, respectively) and log Rank test P value between the high and low expression groups is provided in the column labeled ‘logRankP’. (B) Shown are example Kaplan-Meier survival curves for two ZNFs. The survival data for patients having tumors with the highest (top 30 %) and lowest (bottom 30 %) transcription factor expression is shown; the Log Rank test P value between the high and low groups is indicated. [file 13059_2015_668_MOESM16_ESM.pdf]
